# Biomimetic Model for Electromagnetic Modulation of Cardiovascular Cellular Interactions On-Chip

**DOI:** 10.1021/acsabm.5c00798

**Published:** 2025-07-16

**Authors:** Ana C. Manjua, Fábio F. F. Garrudo, Ana Agostinho, Afonso Gusmão, Paola Sanjuan-Alberte, Frederico Castelo Ferreira, Burcu Gumuscu

**Affiliations:** † Biosensors and Devices Lab, Department of Biomedical Engineering, 3169 Eindhoven University of Technology, Eindhoven 5600 MB, Netherlands; ‡ Department of Brain and Cognitive Sciences, Picower Institute for Learning and Memory, Massachusetts Institute of Technology, Cambridge 02139, Massachusetts, United States; § Instituto de Telecomunicações, Instituto Superior Técnico, Avenida Rovisco Pais, 1049-001 Lisboa, Portugal; ∥ Department of Bioengineering and iBB-Institute for Bioengineering and Biosciences, Instituto Superior Técnico, 72971 Universidade de Lisboa, Av. Rovisco Pais, 1049-001 Lisbon, Portugal; ⊥ Associate Laboratory i4HB—Institute for Health and Bioeconomy, Instituto Superior Técnico, Universidade de Lisboa, Av. Rovisco Pais, 1049-001 Lisbon, Portugal; # IDMEC, Instituto Superior Técnico, Universidade de Lisboa, Av. Rovisco Pais, 1049-001 Lisbon, Portugal; ∇ Eindhoven Artificial Intelligence Systems Institute, Eindhoven University of Technology, Eindhoven 5600 MB, Netherlands; ¶ Institute for Complex Molecular Systems, Eindhoven University of Technology, Eindhoven 5600 MB, Netherlands

**Keywords:** electromagnetic materials, scaffolds, PEDOT: PPS, magnetic particles, coculture, cardiomyocytes, HUVECs, cardiac microenvironment, tissue engineering

## Abstract

Cardiovascular diseases are the leading cause of global mortality. These conditions are associated with cardiac cell death and loss of vascularization, potentially progressing to fatal myocardial infarction. However, the lack of accurate models to simulate the complex cardiac tissue microenvironment and explore alternative therapeutics contributes to heart disease still being regarded as irreversible. In this work, we developed a unique organ-on-chip platform that integrates electrical, magnetic, and mechanical stimulation to replicate the cardiac microenvironment and investigate the impact of electrical and magnetic stimulation on cardiac cell fate. Our micromodel integrated triple stimulating inputs using hybrid stimuli-responsive materials. Electromagnetic scaffolds were obtained by coating with conductive poly­(3,4-ethylenedioxythiophene) polystyrenesulfonate (PEDOT:PSS) electrospun coaxial fibers comprising a polycaprolactone (PCL) shell and a core of gelatin embedded with iron oxide nanoparticles (MNPs). These scaffolds were incorporated in the chip, and the properties and biological effects of these aligned electromagnetic fibers were compared with those of PEDOT:PSS-coated gelatin hydrogels with aligned magnetic particles. In the presence of an external magnetic field, both materials became more hydrophilic. PEDOT:PSS coaxial fibers demonstrated higher electroconductivity (7.9 S·cm^–1^) than the conductive hydrogels (0.83 S·cm^–1^). Induced pluripotent stem cell-derived cardiomyocytes (iPSC-CMs) were successfully cultured on the PEDOT:PSS coaxial fibers, as shown by cell metabolic activity assays over 8 days. Additionally, a 24-h of electric and magnetic combined stimulation significantly enhanced cell viability, with viable cell area increasing from 21% (control) to 54% in the stimulated condition. As proof of concept, we cocultured iPSC-CMs and human vascular endothelial cells (HUVECs) on the materials. Cardiac contraction, which ceased after seeding on the scaffolds, was restored through combined electric and magnetic stimulation and HUVEC culture on-chip. This approach modulates cardiac cell mechanotransduction and offers insights for modeling cardiac tissue, opening future avenues in cardiac repair and remodeling.

## Graphical abstract

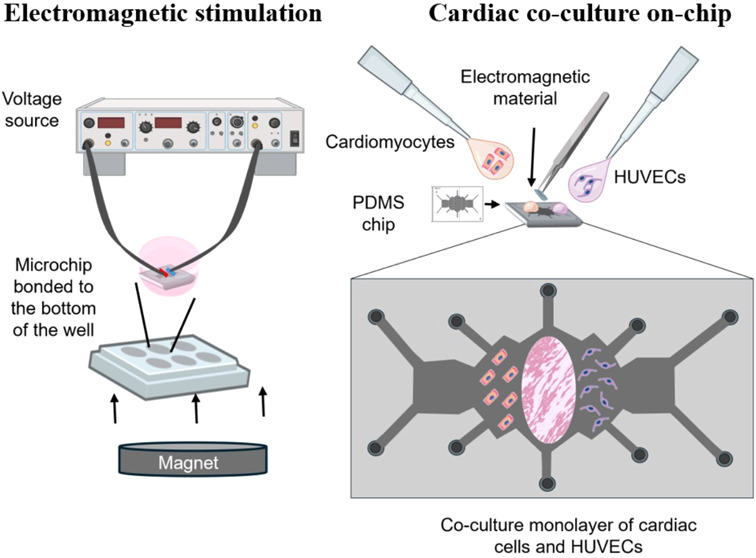

## 1 Introduction

Cardiovascular diseases remain a global burden, continuing to be the leading cause of mortality worldwide and a major contributor to disability despite the major advances in diagnosis and therapeutics made in the last years.^1^ As such, the field requires a better understanding of the underlying biological mechanisms involved in cardiac tissue remodeling and repair.^2^ Cardiac function deterioration occurs naturally with aging, leading to the establishment of cardiac disease even in the absence of pre-existing risk factors. Additional factors, such as poor diet, physical inactivity, tobacco use, and unhealthy lifestyle choices can accelerate this deterioration through complex pathways involved in cell aging and senescence.^3,4^ Moreover, the heart is a complex organ with multiple cell types, such as cardiomyocytes, cardiac fibroblasts, endothelial cells, vascular smooth muscle cells, and immune cells. These cells control cardiac tissue homeostasis and function through a fine-tuned regulation dependent on their complex tissue architecture and interactions.^5^ The microenvironment, particularly the extracellular matrix, serves as a critical mediator of intercellular communication and plays a fundamental role in the development of various cardiovascular diseases.^6^

In the past decades, several tissue engineering approaches have been proposed to mimic the cardiac microenvironment *in vitro.* A special focus was given to mechanical stimulation and optimization of scaffold stiffness.^7,8^ Other strategies include the fabrication of a conductive network in the designated tissue aiming to promote synchronized electrical activity and facilitate cell–cell communication. This was achieved using electroconductive scaffolds composed of carbon nanotubes, graphene and conductive polymers.^9–11^ Electrical stimulation can then be applied, which has shown to influence cellular behavior, particularly in processes such as cell division, proliferation, and gene expression, while also enhancing tissue functionality and maturation.^12^

Magnetic stimulation can also potentially be used to improve cardiac behavior in multicellular systems. Our group has recently explored the effects of magnetic exposure on a coculture of mesenchymal stromal cells (MSCs) and human umbilical vein endothelial cells (HUVECs) in a microfluidic chip.^13^ We reported that magnetic stimulation enhances HUVEC’s survival and endothelialization, highlighting the potential of external magnetic fields to promote vascularization *in vitro*.^13^ However, a challenge remains in exploring whether the magnetic-induced effects on survival and angiogenesis occur in more complex systems, particularly when HUVECs are cocultured with other cell types to replicate the cardiac vasculature, and whether this could facilitate a symbiotic interaction between the cells.

Natural hydrogels, such as gelatin methacryloyl (GelMA), are frequently used for *in vitro* cultures of human cardiac fibroblasts due to their structural similarity to cardiac tissue, which is rich in collagen fibers.^14^ Altering the concentration of GelMA has also been explored to simulate cardiac fibrosis, a condition characterized by a stiffer extracellular matrix primarily composed of collagen types I and III.^15^ All these biomimetic strategies designed to replicate the natural cardiac microenvironment aim to enhance the functionality of synthetic cardiac tissues and their potential for therapeutic applications. Nonetheless, to investigate the underlying mechanisms of heart disease and explore cardiac cell senescence, it is essential to develop scaffolds with tunable properties that can simultaneously promote cell adhesion and deliver a combination of multiple stimulatory factors to the cells.

In the past, authors have described successful approaches to maintain adult myocardial slices (from human and rabbits) *in vitro* with optimal contractility using a preload similar to sarcomere length (2.2 μm) in a large-scale custom-built electroforce culture chamber to provide electromechanical stimulation during 24 h (using human slices) and 72 h (for rabbit slices).^16^ Other researchers developed scaffolds (membranes or hydrogels) with porous or fibrous structures to mimic native microenvironment of cardiac cells.^17,18^ 3D Bioprinting has also been employed to efficiently print *in vitro* tissue constructs with single-cell resolution and high reproducibility.^19,20^

In recent years, *in vitro* cardiac models have been combined with organ-on-chip technologies to obtain heart-on-chip cardiac tissues in a dynamic microenvironment, often integrated with biosensors for real-time screening of physiological features, disease progression and pharmacological responses.^20,21^

Nonetheless, so far none of these cardiac models described in the literature allow us to fully integrate a combination of physiologically relevant stimuli (e.g., electrical, magnetic, and mechanical) in a miniaturized platform. Moreover, their integration with complex models, such as those composed of cardiomyocytes (CMs) and endothelial cells (e.g., human umbilical vein endothelial cells - HUVECs), remains a technical challenge that needs to be overcome. As such, in this work we investigated the combined effects of electrical and magnetic stimulation on the behavior of HUVECs cocultured with induced pluripotent stem cell-derived cardiomyocytes (iPSC-CMs) — as CMs represent the most abundant cell type in the heart, responsible for cardiac function and contractility.^12^ Our hybrid-stimulation approach aims to replicate the electrical microenvironment of the heart while harnessing the benefits of magnetism to promote vascularization in the tissue-like constructs. A key innovation of this work is the development of hybrid stimuli-responsive scaffolds, composed of patterned hydrogels or nanofibers fabricated by coaxial electrospinning for cardiac cell culture (Figure 1A,D). Coupling the scaffolds with an external electric field facilitates the circulation of electrostatic forces within the cell culture medium, promoting the propagation of electrical signals through cell–cell communication and enabling synchronous contractions (Figure 1B,E). Additionally, the incorporation of iron oxide magnetic particles enables these materials to alter their surface properties in response to a magnetic field (Figure 1C,F). The native electromagnetism and mechanosensitive properties of the heart^22^ emphasize the potential of these stimuli to effectively mimic the cardiac microenvironment through the application of combined electrical, magnetic, and mechanical stimulation on-chip.

**Figure 1:**
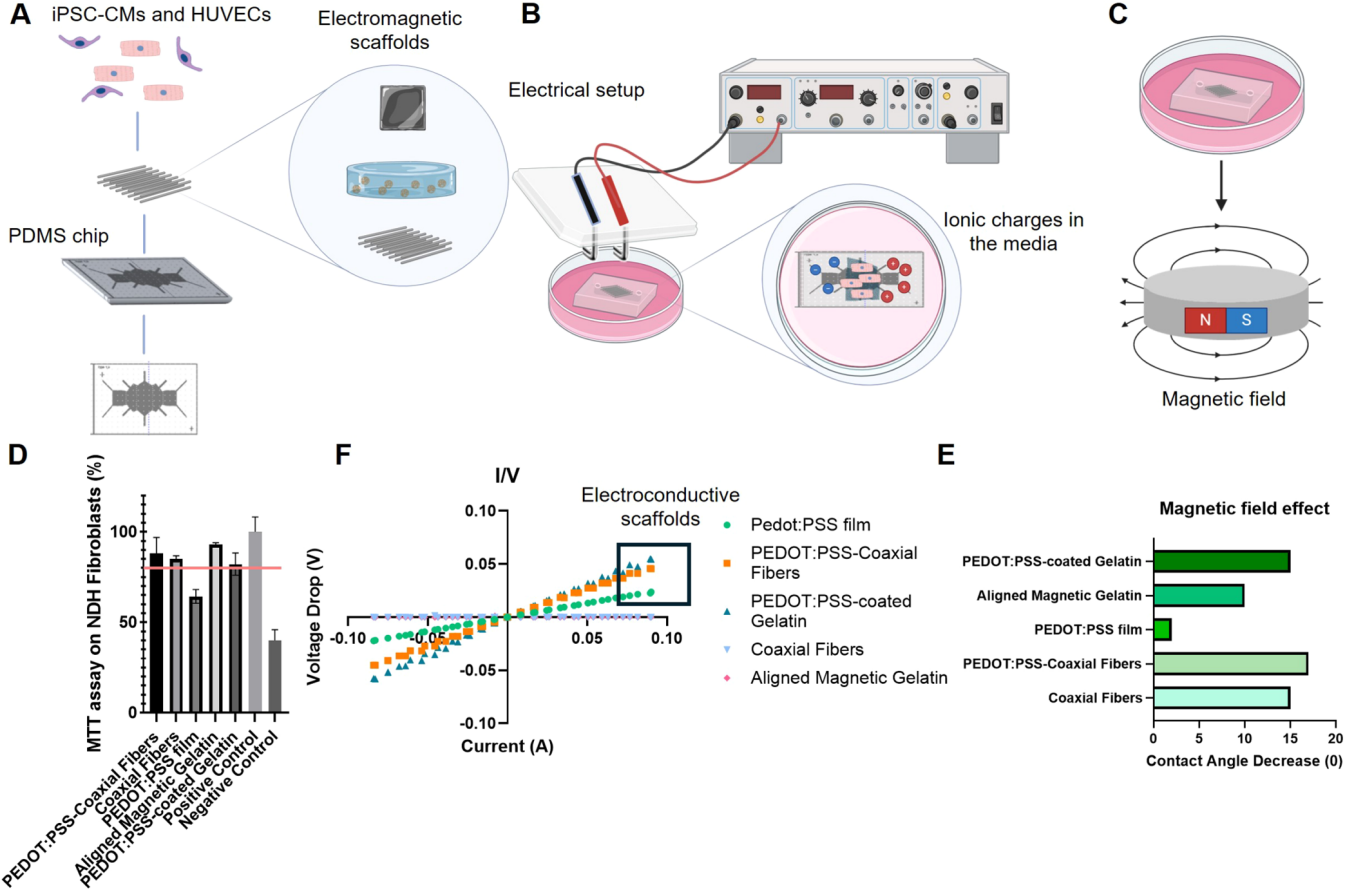
Overview of the protocol used for culturing induced pluripotent stem cell derived-cardiomyocytes (iPSC-CMs) and Human Umbilical Vein Endothelial Cells (HUVECs) on Electromagnetic Scaffolds placed on an organ-on-chip platform and respective characterization. (A) iPSC-CMs and HUVECs are seeded onto electromagnetic scaffolds, including PEDOT:PSS films, electromagnetic hydrogels, and electromagnetic fibers, which are then mounted onto a PDMS chip for support; (B) Electrical stimulation is applied to the cell culture on the scaffold mounted on the chip for a duration of 24 h to induce cellular response; (C) A neodymium magnet is positioned beneath the cell culture for 24 h to investigate the effects of magnetic fields on cell behavior, simultaneously to the electrical stimulation. (D) Biocompatibility assessment of NHD (Normal Human Dermal) Fibroblasts on different scaffolds tested using MTT assay (cell viability exceeding 80% confirms its biocompatibility - Positive control represents NHD Fibroblasts on polystyrene and Negative Control reflects NHD Fibroblasts on latex material). The red line represents the assumed threshold between noncytotoxic (>80%) and cytotoxic (<80%) material; (E) Representative I/V curves of the electroconductive scaffolds tested; (F) Magnetic responsiveness of the scaffolds, as evidenced by an increase in hydrophilicity in response to magnetic field exposure.

To the best of our knowledge, our microdevice represents the first representation of the integrated electrical, magnetic, mechanical, and chemical stimuli in a single organ-on-a-chip platform. This innovation facilitates the modulation of cell mechanotransduction, thereby providing new insights into the precise modeling of cardiac tissue. Understanding the synergistic effects of these combined inputs opens new avenues for advancing strategies in cardiac repair and remodeling.

## 2 Results and Discussion

### 2.1 Fabrication and Characterization of Electromagnetic Composite Nanofibers

Electrospun nanofibers have been widely utilized in cardiovascular tissue engineering applications due to their tunable physicochemical properties.^23^ Moreover, the fabrication of 3D aligned nanofiber scaffolds has been shown to promote a uniform 3D distribution of aligned cardiomyocytes.^24^ In this study, we fabricated coaxial nanofibers with a magnetic core (iron oxide magnetic nanoparticles in gelatin) and a shell of Polycaprolactone (PCL). The magnetic core, necessary to create a local magnetic field, was created by dispersing iron oxide magnetic nanoparticles (MNPs) in a gelatin solution in hexafluoropropanol (HFP). HFP, as a solvent, prevents gelation of gelatin during the electrospinning process, allowing a homogeneous distribution of the MNPs into the nanofiber matrix.

PCL was used as the shell material to encapsulate the magnetic gelatin core and increase the mechanical strength of the fiber mat. PCL has been widely employed in cardiac tissue engineering due to its elasticity, which is compatible with the mechanical properties of cardiac tissue.^25^ The nanofibers were then coated with electroconductive Poly­(3,4-ethylenedioxythiophene) polystyrenesulfonate (PEDOT:PSS) to make the shell electroconductive and suitable for electrical stimulation (Figure 2A). The use of electroconductive substrates has many advantages for cell culture, including enhancing the adhesion, proliferation, and electrophysiological function of cultured iPSC-CMs.^26^ PEDOT:PSS also possess a high conductive efficiency (ZT ∼ 0.42) and has been demonstrated to be suitable for cardiac application. In particular, the fabrication of electroconductive hydrogels has been described to prevent postinfarction arrhythmias and support cardiomyocyte function.^27,28^

**Figure 2:**
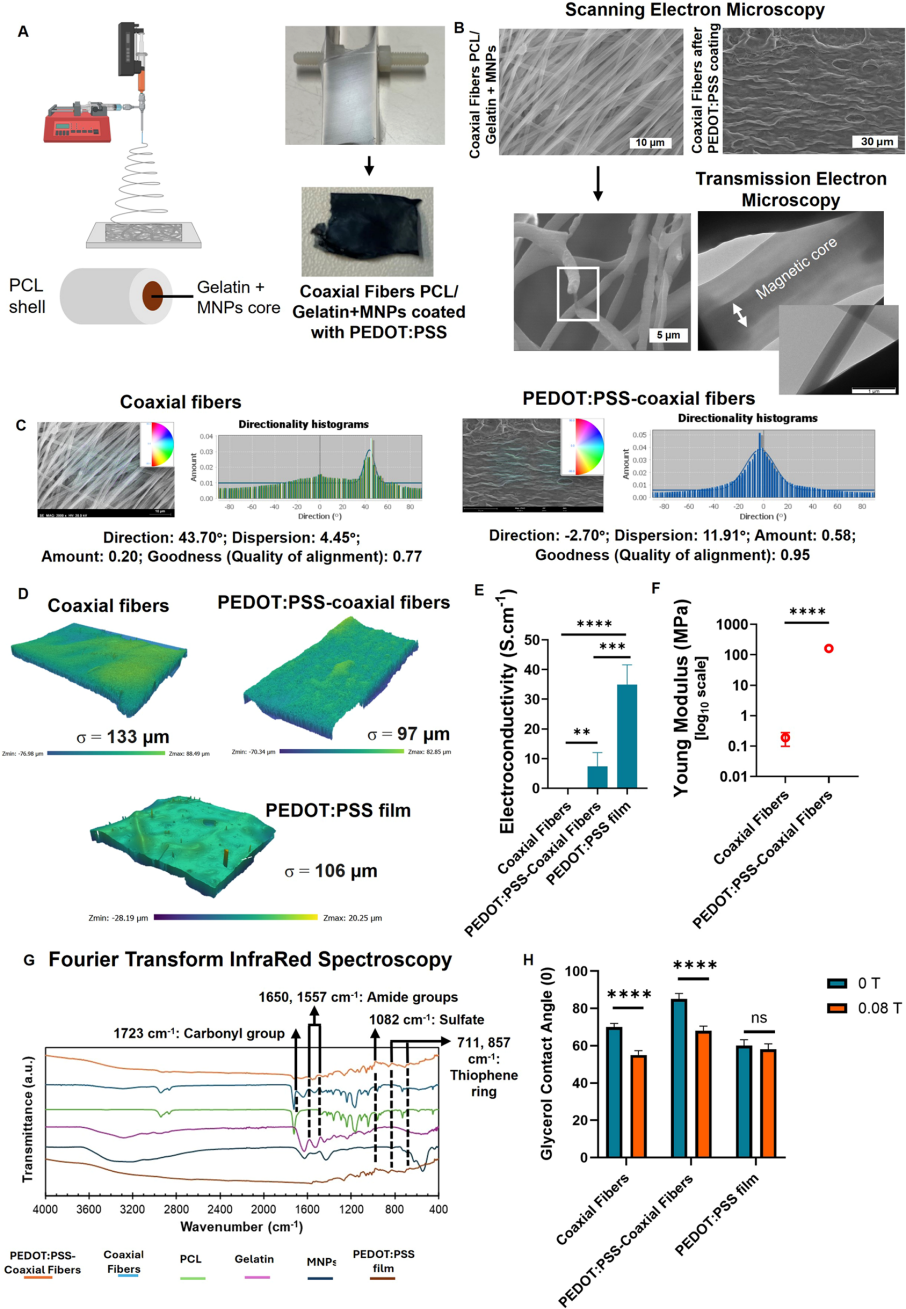
Production and characterization of electromagnetic nanofibers (PEDOT:PSS-Coaxial fibers). (A) Schematic representation of the setup used for the fabrication of coaxial electromagnetic fibers with alignment. (B) Structural analysis of the nanofibers (before and after coating with PEDOT:PSS) using scanning electron microscopy (SEM, surface and cross-section) and transmission electron microscopy (TEM) before PEDOT:PSS coating. (C) Alignment results for coaxial fibers and PEDOT:PSS-coaxial fibers using the directionality plugin (Fourier components analysis) in Fiji. (D) Surface profile and roughness analysis of the nanofibers. (E) Quantification of the electroconductivity of the nanofibers in comparison with a standard PEDOT:PSS film. (F) Mechanical characterization of the fibers using uniaxial tensile technique. (G) Chemical characterization of the fibers through Fourier-Transform Infrared Spectroscopy (FTIR). For comparison purposes, PCL, Gelatin, MNPs and a film of PEDOT:PSS were also analyzed. (H) Glycerol contact angle was determined by the sessile drop technique in the presence and absence of a magnetic field to test magnetic responsiveness of the fibers. PEDOT:PSS film without magnetic content was used as a negative control.

The fabricated noncoated coaxial fibers show a good alignment and have an average diameter of 250.2 nm (with a standard deviation error of 41.71 nm and a standard error of mean of 4.82 nm, *n* = 75) (Figure 2B). Additional images of the fibers are shown in Figure S2. The histogram for coaxial fiber diameter can be found in Figure S2A. After coating with PEDOT:PSS, the average fiber diameter increases to 361.2 nm (with a standard deviation error of 149.8 nm and a standard error of mean of 18 nm, *n* = 70) due to fiber heterogeneity, as can be observed in the SEM images and histogram from Figure S2B. The scaffold topology after coating is affected by the PEDOT:PSS coating across the fiber mesh and by the PEDOT:PSS cross-linking temperature at 50 °C, which caused molten fibers in some specific regions. Random fiber orientation after coating can also be identified in some regions, as seen in Figure S2B as a consequence of the thermal treatment, which is known to cause: (i) fiber contraction from the entropic relaxation of the molecules and (ii) shrinkage from chemical reactions, ultimately leading to loss of orientation.^29^ Nonetheless, the alignment after coating is mostly preserved across the scaffold area (Figure 2B). Analysis of the orientation of the fibers, before (43.70°, with a quality of alignment of 0.77 out of 1) and after coating (−2.70°, with a quality of alignment of 0.95), was determined with the Fiji Directionality plugin (Figure 2C), further confirming that the scaffolds retain their primary orientation following the coating process. Therefore, the observed shrinkage effect was not considered to undermine the objectives of this study. The MNPs within the fiber core were observed by SEM, revealing an average clustered size of 0.63 μm (Figure S2C), a size that enhances the magnetic response of the coaxial fibers. The coaxial structure of the fibers was confirmed by TEM (Figures 2B and S3), where differences in higher density inside each fiber appear with a darker contrast at the core of the fiber composed of noncrosslinked gelatin with magnetic particles in liquid form (Figure 2B). Optical analysis of the fibers surface and roughness allowed us to confirm the slight shrinkage in the PEDOT:PSS-coated fibers induced by the thermal treatment in comparison with the coaxial fibers before coating (Figure 2D). The surface profile is also compared to the smoother surface of a film of PEDOT:PSS, also subjected to thermal treatment during crosslinking (Figure 2C), highlighting that the PEDOT:PSS-coaxial fibers share a surface profile closer to the coaxial fibers than to a film of PEDOT:PSS.

The electroconductivity of the fibers was evaluated using a four-point probe technique (Figure 2E). The results confirmed that the coaxial fibers lacked conductivity, whereas the PEDOT:PSS-coated coaxial fibers exhibited conductivity values of the same magnitude order (∼10 S·cm^–1^) of a standard DVS cross-linked PEDOT:PSS film (∼40 S·cm^–1^). These values are within the range of conductivity of the ventricular muscle, blood or skeletal muscle, ranging between 3 and 60 S·cm^–1^.^30^

Due to the liquid core of these fibers, it was important to determine the mechanical resistance (Figure 2F). Tensile mechanical testing was performed to measure the elasticity modulus (Young’s Modulus) of the fibers before and after the coating. It was observed that the mechanical resistance of the fibers increased from 0.2 to 162 MPa after coating them with PEDOT:PSS. Even so, the PEDOT:PSS-coaxial fibers still allowed for a deformation of around 18% before breaking (Figure S4). These values are much higher than the values found in the literature for the Young’s Modulus of diastolic adult human myocardium (in the range of 8–15 kPa).^31^

However, the Young’s Modulus of the human heart can range up to 50 MPa.^28^ The higher values we obtained with the coaxial fibers is related to the natural stiffness of the PCL being higher than the gelatin, in the case of the coaxial fibers, which is further increased by the addition of the PEDOT:PSS coating. Nonetheless, PCL was specifically chosen to provide mechanical resistance, otherwise it would be very difficult to handle the aligned fibers during the experiments without breaking them.

Dynamic scanning calorimetry (DSC) results for the coaxial fibers before coating with PEDOT:PSS show a fusion temperature consistent across the cycles (56.5 °C in Cycle 1, 56.3 °C in Cycle 3, 56.4 °C in Cycle 5) (Table S1 and Figure S1). This stability indicates that the amorphous phase of the material is stable and does not undergo significant thermal degradation or structural changes across the cycles. Due to minimal variation in this temperature (0.2 °C between the highest and lowest values) suggests good thermal stability. The crystallization temperature with a 32.8 °C peak was also consistent across cycle 2 and cycle 4, representing a reproducible thermal transition, likely related to secondary crystallization. The consistency of the temperature across cycles indicates a stable and reversible transition. The results for the PEDOT:PSS-coaxial fibers were very similar, with a similar transition temperature around 32 to 33 °C. As expected, this similarity between the two samples suggests that both materials may have similar properties or behavior at lower temperatures.

We also chemically characterized the fibers to understand the contribution of the MNPs to fiber composition. FTIR results shown in Figure 2G highlight that the PEDOT:PSS-coaxial fibers have a profile almost identical to the PEDOT:PSS film, clearly showing a peak at 1082 cm^–1^ characteristic to sulfate present in PEDOT:PSS.^32^ The spectra is dominated by the signal of the PEDOT:PSS coating, with visible characteristic low-intensity peaks (e.g., 711 and 857 cm^–1^, thiophene ring).^33–35^ The PEDOT:PSS-coaxial fibers also show low-intensity peaks typical to PCL (e.g., 1723 cm^–1^, carbonyl group) and gelatin (e.g., 1557 and 1650 cm^–1^, amide groups), but not of MNPs (e.g., 550 cm^–1^) due to its encapsulation.^33–35^ The coaxial fibers, however, showed a combination of the peaks from gelatin and the PCL, evidencing that the PCL shell was not predominant in the chemical surface of the fibers and there was also contribution from the gelatin. The peaks from the MNPs cannot be observed, which may be attributed to the fact that these peaks were low in intensity and easily masked by other unspecific peaks. Since the different layers in the fibers were separated, bond breakage was not expected. As a result, the peaks maintained their transmittance without any increase or decrease. Likewise, it is suggested that the original chemical structure is preserved in the conductive fibers, once again confirming the independent layers within the fibers, as we did not observe new peaks in the FTIR of the conductive materials in comparison with the isolated materials (PCL, Gelatin, MNPs, PEDOT:PSS). Finally, it was important to assess whether the presence of the MNPs could still provide magnetic responsiveness in both the coaxial fibers and the PEDOT:PSS-coaxial fibers despite being in the core of the fiber and not showing up in FTIR results.

The glycerol contact angle of the fibers was measured both with and without a magnet placed beneath them for 5–10 min prior to measurement (Figure 2H). This was done to allow for the movement of MNPs mixed within the noncrosslinked gelatin, potentially altering the material’s surface roughness. In fact, the results show a decrease of contact angle in the range of 10–15° when a magnetic field of 0.08 T is applied on both fibers. As expected, no effect is observed in the control PEDOT:PSS film as there is no magnetic compound in this film. These results demonstrate an increase in the hydrophilicity of the conductive fibers due to the exposure to the magnetic field, which is a parameter known to be better suited for cell culturing as hydrophilic surfaces typically enhance cell attachment and improving the regulation of immunomodulation.^36^ The magnetic responsiveness of the fibers also confirms the ability of the MNPs to create an impact on the surface of the fibers despite their inner location.

### 2.2 Fabrication and Characterization of Patterned Electromagnetic Hydrogels

Hydrogels have long been considered the gold standard in cell culture, including in cardiac cell culture.^37^ Hence, we compared the fabricated conductive fibers with gelatin-based hydrogels, specifically designed with a patterned topography to mimic the effects of aligned fibers. We aligned MNPs to a magnet during gelation process to control the size of the stripes based on the magnetic field density (Figure 3A). We could mimic the fiber geometry on a hydrogel using a 3% concentration of aligned MNPs, which was maintained throughout the experiments. The magnetic alignment and consequent surface roughness were preserved even after PEDOT:PSS coating, as previously employed with the fibers (Figure 1A). Interestingly, the surface roughness stemming from the particle alignment was not observed macroscopically after glutaraldehyde crosslinking, which was also confirmed by SEM images (Figure 3B), but it becomes noticeable again after coating with PEDOT:PSS and DVS thermal crosslinking. We attribute this occurrence to the shrinkage promoted by thermal treatment.

**Figure 3:**
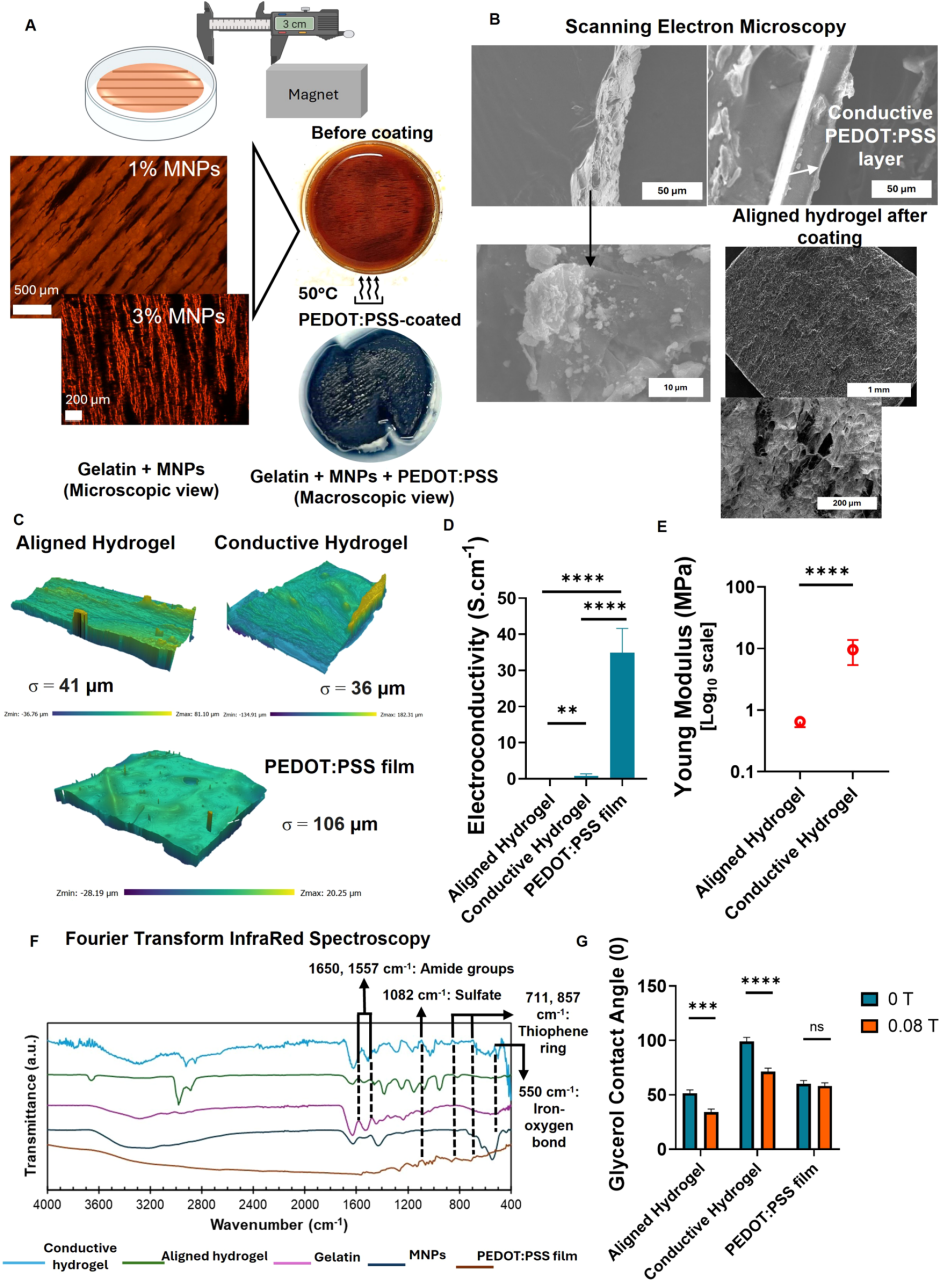
Characterization of electromagnetic hydrogels (conductive hydrogel). (A) Setup for the fabrication of hydrogels with embedded magnetic iron oxide MNPs for an aligned topography and microscopic and macroscopic view of the produced aligned and conductive hydrogels. (B) Structural analysis of the aligned hydrogels (before and after coating with PEDOT:PSS) using SEM (surface and cross-section). (C) Surface profile and roughness analysis of the hydrogels. (D) Quantification of the electroconductivity of the hydrogels in comparison with a standard PEDOT:PSS film. (E) Mechanical characterization of the hydrogels using compression technique. (F) Chemical characterization of the hydrogels through FTIR. For comparison purposes, Gelatin, MNPs and a film of PEDOT:PSS were also analyzed. (G) Glycerol contact angle was determined through the sessile drop technique in the presence and absence of a magnetic field to test magnetic responsiveness of the hydrogels. PEDOT:PSS film without magnetic content was used as a negative control.

SEM images demonstrated particles on the cross section of the hydrogel, where a distinctive layer of conductive PEDOT:PSS is observed in Figure 3B. Optical analysis of the dried surface of the hydrogel (Figure 3C) showed protrusions on the surface of the conductive PEDOT:PSS coated hydrogel, that were not observed on the pristine hydrogel; with both samples displayed surface patterns in comparison to the smooth surface of the standard PEDOT:PSS film. This dehydrated hydrogel was also thinner (around 40 μm) in comparison to the fibers and to the PEDOT:PSS film (around 100 μm). However, the decreased water content did not represent the suitable conditions for cell culturing. The hydrated state of the hydrogel had a thickness ranging from 100 to 150 μm, while the PEDOT:PSS-coated hydrogel, due to the thermal treatment, reached only 70 μm under hydration afterward. The electroconductivity of the materials was tested using the four-point probe method (Figure 3D) and it was found that the conductive hydrogel has a much lower electroconductivity (approximately 0.8 S·cm^–1^) than the PEDOT:PSS-coaxial fibers, thus closer to the biological values discussed in the previous section.

Next, we investigated the mechanical properties of the hydrogels. The Youngs Modulus of the hydrogels was demonstrated to be closer to the previously reported results for the biological tissues,^31^ with the conductive hydrogel having a Young Modulus of around 9.6 MPa, and the aligned hydrogel having 650 kPa (Figure 3E).

The DSC analysis (Table S1) for the materials resulted in similar conclusions to those found for the fibers. DSC results indicated stability throughout the different cycles tested, while the chemical characterization through FTIR (Figure 3F) showed that the profile of the conductive hydrogel was a mixture of all the present compounds. We observe a distinct peak from the MNPs at 550 cm^–1^, and a characteristic group of gelatin peaks centered at 1650 cm^–1^. The sulfate peak characteristic from the PEDOT:PSS at 1082 cm^–1^ is present, but it is slightly masked by the peaks from the gelatin around the same wavelength. Nonetheless, unlike what was observed for the fibers, the profile of the conductive hydrogel was not dominated by its PEDOT:PSS composition, which could partly explain the significantly lower electroconductivity in comparison to the fibers.

Next, the glycerol contact angle measurements revealed increased magnetic responsiveness in comparison to the conductive fibers due to a decrease of contact angle around 20° under magnetic exposure. This can be attributed to the absence of the PCL layer in the hydrogels. Nevertheless, the contact angle of the conductive hydrogel, despite the decrease in the presence of the magnetic field, was still higher than 70°, making this material less ideal for cell culturing.

### 2.3 Effect of Combined Electrical and Magnetic Stimulation on Cardiac Cell Behavior On-Chip

The iPSC-CMs used in this study were derived from human iPSCs following the protocols available in the literature.^38^ The purity of the iPSC-CMs was determined to be around 73.6% through the immunostaining of iPSC-CMs with a monoclonal antibody against cardiac Troponin I (Figure S5A), following the protocol described in the Section 4.14. The functionality of the iPSC derived CMs was first evaluated by the observed beating properties on day 10 of differentiation (Supporting Movie S1, Figure S5A). The iPSC-CMs were also evaluated on day 20 prior to cell culture. Their contractility profile was obtained using MuscleMotion (Figure 4A) and Myocyter ImageJ plugins, extensively used in the literature and reliable tools for this analysis.^39,40^ Myocyter identifies single cells within the monolayer and provides their contraction profile, allowing to combine multiple cells in the same graph and providing information related to the beat times (average 0.99 s), frequency (11.7 s^–1^), amplitudes (23.4 au), peak times (0.5 s), systole relaxation (0.3 s) and diastoles relaxation (0.2 s) (Table S2). The normalized contraction speed obtained from the MuscleMotion tool shows consistent beating and synchronization within the iPSC-CMs. These results assured us of the quality of iPSC-CMs to be cultured in the microchips.

**Figure 4:**
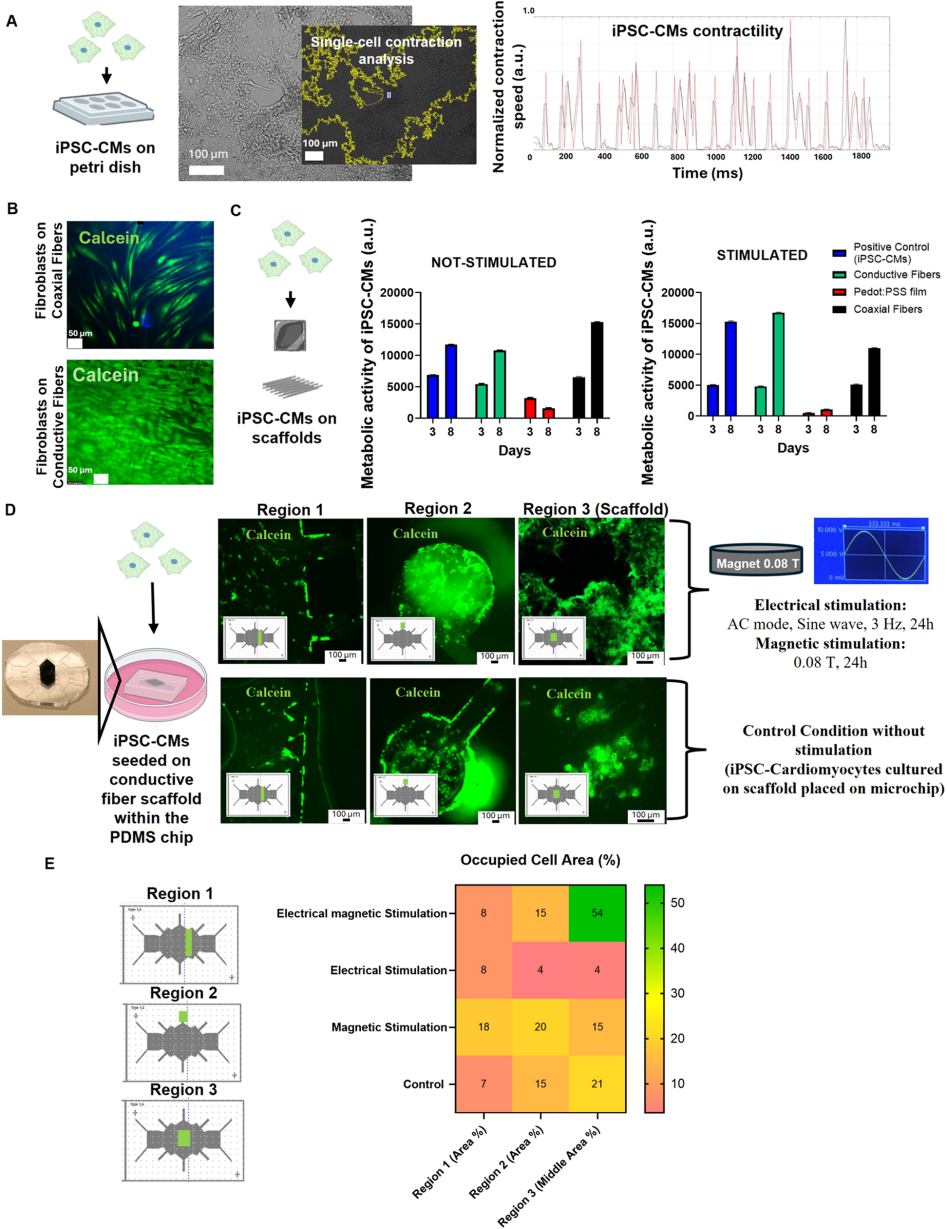
Characterization of the combined effect of the electromagnetic scaffold and external stimulation on the iPSC-CMs seeded on-chip. (A) Characterization of the contractility behavior of iPSC-CMs prior to seeding on the scaffold and stimulation. (B) Biocompatibility of the scaffolds (Coaxial fibers and conductive fibers scaffolds) on dermal fibroblasts (NHDF cell line) and morphological changes (*n* = 3). (C) iPSC-CMs metabolic activity on the scaffolds, measured by fluorescence intensity of Alamar blue, from day 0 (after cell seeding and before adhesion) until day 8 (including conditions with electrical stimulation on day 7 and comparison to the nonstimulated condition) (*n* = 3). (D) iPSC-CMs distribution on different areas of the chip after electrical and magnetic stimulation (on day 8) in comparison to the control condition without stimulation (*n* = 3). (E) Quantification of the occupied area (%) of viable cells in the three analyzed regions for all the tested conditions using ImageJ (*n* = 3).

Prior to the seeding of iPSC-CMs on the conductive fibers/hydrogel placed on the chip, cytotoxicity and biocompatibility analyses of the different scaffolds were performed. The biocompatibility of these materials was evaluated using human dermal fibroblasts (NHDF) cell line.

The results of the MTT indirect cytotoxic assay (Figure S6A1) indicated the cytotoxic behavior of the conductive hydrogel and the PEDOT:PSS film, with a viability below 80%. For the aligned hydrogel, the final viability was 85%, which is still at the limit of the cytotoxicity range. This effect could potentially be controlled by further cleaning steps to remove any left toxic molecules that could lixiviate to the culture media. However, it could compromise the surface patterning. The direct contact test allowed us to conclude that the assessed materials are not cytotoxic, as no inhibition halos were observed in the three conditions: aligned hydrogel, conductive hydrogel and PEDOT:PSS film, and negative control (Figure S6A2).

By seeding the fibroblasts on top of the scaffolds, we observed the alignment of the viable fibroblasts on both coaxial fibers and particularly on PEDOT:PSS-coated fibers (Figure 4B) while the fibroblasts seeded on the hydrogels spontaneously migrated inside the hydrogel, which became encapsulated as can be seen in Figure S6B. The fact that the fibroblasts migrated toward the inner part of the hydrogel prevented the alignment seen on the fibers and fewer fibroblasts were observed on the conductive hydrogel.

The biocompatibility of PEDOT, doped or not with PSS, has been subject of controversial discussion throughout the years. While some works claim the noncytotoxic properties of PEDOT:PSS,^41,42^ others acknowledge the issues in biocompatibility of this conductive material.^43–47^ For this reason and due to the promising noncytotoxic results of the fibers, in this work the iPSC-CMs were cultured (on day 21 of differentiation) and tested on the fabricated fibers.

Initially, the iPSC-CMs were seeded on a small density of 20,000 per cm^2^ on the fibers over 8 days of experiment and their metabolic activity was evaluated using Alamar blue kit (Figure 4C). On day 7, for a set of conditions, the samples were subjected to electrical and magnetic stimulation using a custom-built electrode setup connected to a voltage source and a magnet beneath the cell plate. The results showed an increase in metabolic activity from day 3 to day 8, at values of 7500 to 1300 for the positive control (iPSC-CMs on polystyrene without scaffold) without stimulation and from 5100 to 15500 when subjected to electrical and magnetic stimulation. A similar result was obtained for the iPSC-CMs seeded on the PEDOT:PSS-coated coaxial fibers scaffold, while nonsignificant differences were observed for the noncoated coaxial fibers in the conditions with or without stimulation; even though an increase in metabolic activity was identified from day 3 to day 8 in both cases. For the iPSC-CMs seeded on the PEDOT:PSS film, the decrease in metabolic activity is suggestive of cell death, probably due to the high conductivity of the material coupled with the electrical stimulation.^47^ iPSC-CMs are not prone to proliferate after differentiation, still our results show increase of metabolic activity measured by Alamar blue over culture time. One can hypothesize that the iPSC-CMs might have undergone some type of hypertrophic growth due to the increase in their cellular, metabolic activity, typically associated on cardiomyocytes to an increase in cell size and protein content in response to stimuli.^48^ This increase can also be attributed to the presence of other cardiac progenitors within the cardiac cultures.

The electrical stimulation setup used in this experiment have been used within our group to promote cardiac cell alignment and improve contractility (Figure S7).^46^ However, in the current setup, electrodes were not in direct contact with the scaffolds, thus resulting in a lower voltage reaching the cells. This effect induces ionic stimulation through the cell culture media while potentially enhancing transmission of electrical signals between the cells. Consequently, we decided to increase the voltage to a minimum 0 V and maximum 10 V and frequency to 3 Hz, which is above the usually recommended values for direct electrical stimulation of cardiac cell cultures (up to 8 V and 1 Hz).^47^ However, a similar setup has been applied in another study using bipolar stimulation with platinum electrodes (width 10 ms) and a voltage ranging from 2 to 40 V (occasionally reaching 50 V) in a large-scale chamber containing cell culture media and myocardial tissue slices.^16^ Using this setup, the authors were able to efficiently maintain the contractility, structure, functionality and transcriptional properties of the slices for 24 h using human slices and 5 days using rabbit myocardial slices. Hence, this approach was shown to significantly prolong the culture of adult cardiac tissue *in vitro*.

To characterize the morphology, distribution and viability of iPSC-CMs, these cells were seeded on the PEDOT:PSS-coated coaxial fibers scaffolds integrated on the PDMS chip and subjected to electrical and magnetic stimulation (PDMS was coated with a thin film of gelatin to improve cell adhesion). The same procedure was repeated for the positive control (iPSC-CMs seeded on the scaffold placed on the PDMS chip without stimulation) (Figure 4D) and for the single electrical and single magnetic stimulation used as additional controls of the experiment (Figure S8).

It is possible to distinguish between the areas where iPSC-CMs were cultured on top of the gelatin-coated PDMS and on the surface of the scaffold (in the middle compartment) thanks to the geometry of the chip. Calcein-AM staining images show differences in viability of iPSC-CMs between the different areas of the chip in Figure 4D (Region 1, 2 and 3). Region 1 and 2 represent iPSC-CMs seeded on the gelatin-coated PDMS while Region 3 represent the iPSC-CMs seeded on top of the PEDOT:PSS-coaxial fiber scaffold. The images suggest the presence of a higher number of viable iPSC-CMs in the condition exposed to electrical and magnetic stimulation in the middle region (Region 3). As the conductive scaffold is only located in the middle area (Figures 4D and S7), the increased confluency of iPSC-CMs in that region seems to be related to the combined effect of the conductive scaffold with the stimulation.

To quantify the percentage of viable iPSC-CMs occupying the different regions of the chip across the different stimulation conditions, a heat map of the occupied cell area (%) was obtained (Figure 4E), confirming that the combined electrical and magnetic stimulation in the Region 3 of the chip leads to 54% of occupied area with viable iPSC-CMs. In comparison, the control for the same region resulted in only 21% of area occupied by iPSC-CMs while the remaining conditions showed results below 20% for the remaining regions.

Interestingly, the analysis of the conditions for single magnetic stimulation and single electrical stimulation (Figure S8), also considering the respective assessment of the occupied area (%) of viable iPSC-CMs (Figure 4E), indicates that the magnetic field application seems to play a role in increasing the viability of the iPSC-CMs. Note that these results were obtained using the iPSC-CM culture alone. We previously reported a similar effect in the increased viability of MSCs and HUVECs under a static magnetic field of 80 mT,^49,50^ with MSCs showing signs of alignment, proliferation, increased viability and metabolic activity. This effect was also observed in another study using macrophages.^51^ Still, in this study, there is no observation of alignment. Figure S8 shows poor viability and low cell density of iPSC-CMs under single electrical stimulation in all regions in comparison with the condition exposed to magnetic stimulation and to the control condition. This effect suggests that the electrical forces propagate through the cell culture media due to ionic stimulation, affecting the cells of the entire area of the microdevice. It is possible that the magnetic field counteracts the toxic effects of electrical stimulation for the electrical and magnetic condition, thereby improving cell viability under these conditions.

The fact that the fluorescence of Calcein and the % of occupied cell area in Region 1 and 2 do not evidence significant differences between the electrical, magnetic, and control conditions, highlights the contribution of the scaffold to the distinct effect observed in the Region 3 (middle area) for the electrically and magnetically exposed condition.

Throughout this particular experiment, it was also observed that the iPSC-CMs were not able to beat in any of the conditions tested, despite several works showing improved contractility under electrical stimulation.^46,47^ Furthermore, Figures 4D and S8 demonstrate that the iPSC–iPSC-CMs have a rounded morphology atypical for healthy CMs, which should display a rod-shaped morphology. A similar morphology has been discussed before^52^ after CMs had been encapsulated in bioinks for 3D bioprinting. It is therefore possible to consider that the iPSC-CMs feel entrapped by the scaffold patterning and prefer to cluster with each other for survival, thus forming the shape observed in Figure 4D.

Regarding the Regions 1 and 2, PDMS is hydrophobic and possesses low cell affinity,^37^ which may have affected the morphology of the iPSC-CMs despite the thin coating of gelatin on its surface. A coating with laminin could potentially improve the results obtained for the positive control due to the specific biochemical cues that laminin provides. However, we decided to coat the PDMS with gelatin to maintain consistency with the composition of the scaffold.

### 2.4 Co-Culturing HUVECs and Cardiomyocytes under Stimulation to Trigger Contractility

Cardiomyocytes have been described to benefit from coculture with other supporting cells, including cardiac fibroblasts^53^ and endothelial cells.^54^ An interesting work^54^ revealed that the incorporation of endothelial cells during iPSC-derived cardiomyocyte differentiation promoted accelerated maturation, increasing their size and the expression of sarcomere, ion channel genes and proteins. In extension, another study has investigated an improved functionality of CMs cultured with endothelial cells indicating advanced CM maturation in a cardiac tissue model.^55^

In our proof of concept model, iPSC-CMs were cultured on day 1 on the chip with PEDOT:PSS-coated fibers at a higher cell density of 1,000,000 cells. The scaffold on the chip was preconditioned with iPSC-CM cell culture media on day 0 for 24 h before the seeding of the iPSC-CM cells. To more accurately mimic the dynamics of the cardiac tissue and to restore the contractility of the iPSC-CMs, HUVECs (cell density of 2,500,000 cells) were cocultured on day 3 within the monolayer of iPSC-CM cells (Figure 5A). The strategy to seed HUVECs after the iPSC-CMs was related to the fact that cardiomyocytes are highly sensitive to their microenvironment and typically require time to attach to the substrate. Therefore, seeding HUVECs first could interfere with the formation of a healthy monolayer of the iPSC-CMs. Furthermore, HUVECs, once seeded, are prone to migrate, proliferate and form a network. Hence, this strategy should allow the endothelial cells to integrate into the microenvironment without disturbing the initial architecture of the iPSC-CMs and promote the formation of endothelial networks around the cardiac cells.

**Figure 5:**
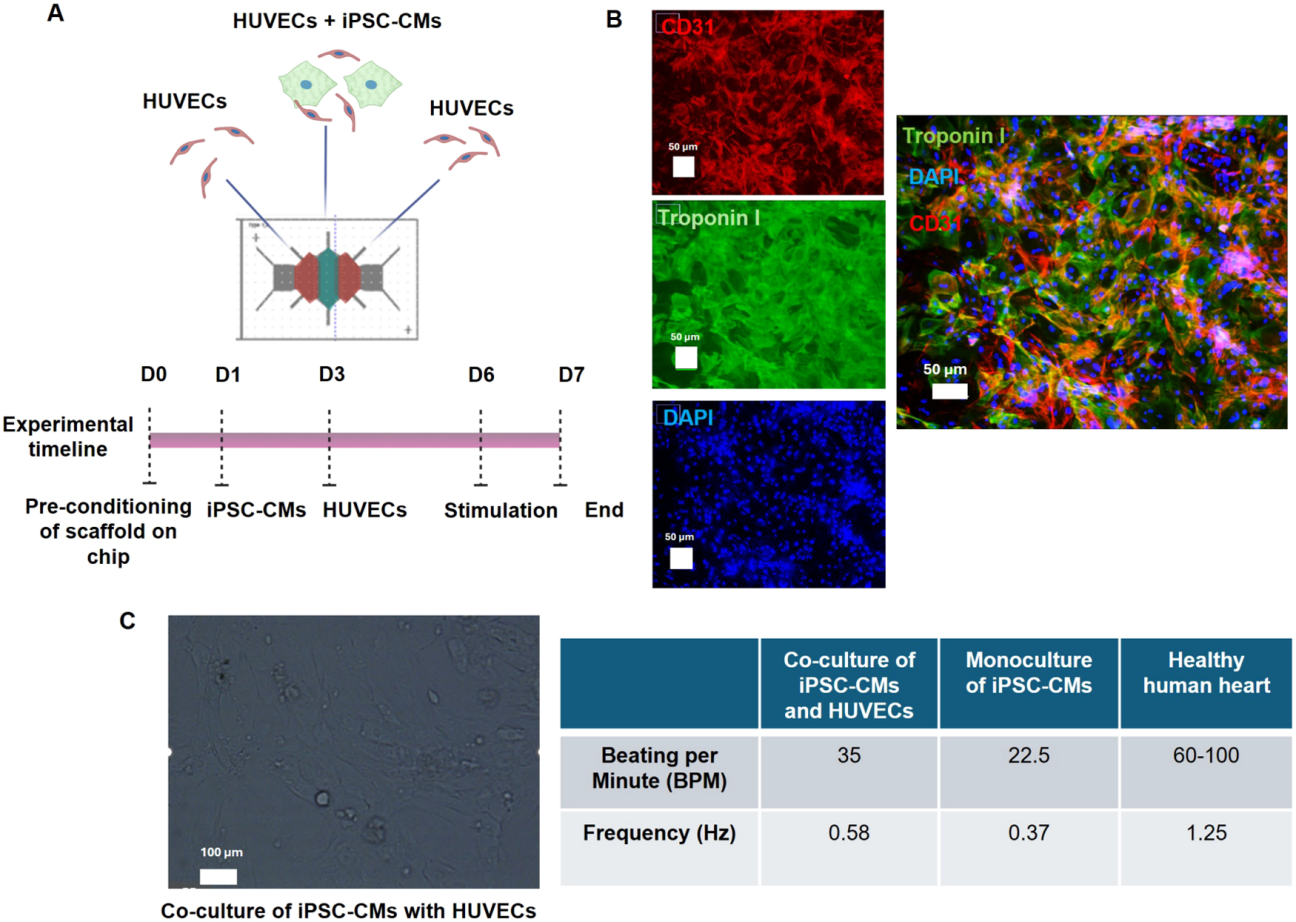
Characterization of the coculture of iPSC-Cardiomyocytes with HUVECs on-chip on day 7. (A) Schematic representation of the experiment. (B) Immunofluorescence images of the HUVECs (targeted with CD31 antibody) and iPSC-CMs (targeted with Troponin I). (C) Contractility analysis of the coculture of iPSC-CMs and HUVECs in the middle region of the chip and comparison with a monolayer of iPSC-CMs.

The system was subjected to electrical and magnetic stimulation on day 6, and after 24 h, the cells were fixed and immunostained to observe their morphology and distribution on the conductive scaffold. HUVECs were targeted by CD31 (red) and the iPSC-CMs were targeted with Troponin 1 - TNNi3 (green). The images show an interconnection between the cell types in a desired tissue-like vascular network architecture (Figure 5B). In the coculture, iPSC-CMs also preserved their characteristic rod-shaped morphology instead of the unhealthy morphology observed in Figure 4D. The cells were filmed before fixation to assess signs of contractility on the final day of the experiment (day 7) (Figure 5C). iPSC-CMs beating was detected in multiple regions of the monolayer of HUVECs with iPSC-CMs, appearing synchronized and beating as a whole construct with a distinct morphology from the previous monocultured iPSC-CMs (Supporting Movie S2. Monoculture of CMs and Movie S3. Coculture of iPSC-CMs with HUVECs). As done previously for the monoculture of iPSC-CMs after differentiation, the contractility observed in the cocultured layer of cells was analyzed by Musclemotion and Myocyter tools (Figure S9). Despite the clear observation of contractile movement in the video, the imaging tools could not identify the morphology of the beating cardiomyocytes within the coculture with HUVECs. Therefore, the videos from the monoculture of iPSC-CMs and the cocultured cells were manually analyzed. It was concluded that the iPSC -CM/HUVEC cocultured on the PEDOT:PSS coated coaxial scaffold, placed on the chip and electrically magnetic stimulated, induced a Beating Per Minute (BPM) of 35 with an average frequency of 0.58 Hz against a BMP of 22.5 and a cardiac frequency of 0.37 Hz for the initial monoculture of iPSC-CMs cultured on the polystyrene cultured plates before seeding on the scaffolds. The healthy human heart has an average BMP ranging from 60 to 100 and a frequency of 1.25 Hz;^56^ therefore, the values from the coculture are closely related to the values from human physiology. The cardiac contractility is regulated by changes in intracellular calcium concentration, where the calcium concentration should be sufficiently high in systole and low in diastole in a healthy individual.^57^ In heart hypertrophy, a combination of events triggered by the mechanical stretch of cardiac myocytes leads to the stimulation of myocardial angiogenesis as a survival mechanism to prevent hypoxia and preserve cardiac contractility.^58^ While VEGF-A, the key molecule to trigger angiogenesis in endothelial cells, activates CMs by promoting morphogenesis, and inducing contractility, VEGF-A is also secreted by CMs during inflammation, mechanical stress and cytokine stimulation,^59^ thus triggering an angiogenic behavior in endothelial cells and creating an important symbiotic role between these two cell types to promote heart remodeling. Likewise, in this work we observed how the addition of HUVECs to iPSC-CMs cultured on a conductive scaffold in an organ-on-chip platform, coupled with electrical and magnetic stimulation, resulted in a HUVEC-CMs interaction leading to a restored and improved contractility and iPSC-CMs morphology.

## 3 Conclusions

This work pioneers the integration of electrical and magnetic stimulation in a cardiac organ-on-chip platform to modulate cardiac cell behavior. The fabricated composite materials demonstrated high electromagnetic performance, superior electroconductivity compared to standard conductive films of PEDOT:PSS, and magnetic-responsive behavior, as evidenced by increased hydrophilicity under magnetic field exposure. Overall, PEDOT:PSS-coated coaxial fiber scaffolds outperformed electromagnetic hydrogels, exhibiting higher electroconductivity (7.9 S·cm^–1^) and physiologically relevant mechanical properties, including stretchability similar to native cardiac tissue. Differentiated CMs showed greater affinity for the conductive fibers compared to patterned electromagnetic hydrogels. The electrical and magnetic setup promoted improved cardiac cell adherence and viability. The addition of HUVECs to the culture of iPSC-CMs on the chip allowed to restore the characteristic beating properties of the iPSC-CMs, which had ceased after seeding on the electromagnetic scaffolds. Overall, this electroconductive model holds significant potential for future applications in cardiac tissue remodeling, disease progression studies, and regenerative repair.

## 4 Experimental Section

### 4.1 Synthesis of Magnetic Nanoparticles

The magnetic nanoparticles (MNPs) were synthesized using a coprecipitation method.^60^ A 25 mL solution of 0.35 M FeCl_2_ (3.58 g of FeCl_2_.4H_2_O) and 0.72 FeCl_3_ (9.73 g of FeCl_3_·6H_2_O) (Sigma-Aldrich) was produced and agitated at room temperature until dissolved. In a nitrogen environment, a 1.0 M NH_4_OH (Sigma-Aldrich) solution was prepared in Milli-Q ultrapure water and stirred continuously at 1250 rpm. The iron salt solution was then added drop by drop using a flow rate of 5.0 mL·min^–1^ with a pump. MNPs were formed spontaneously due to the coprecipitation of the two iron slats in media with high pH. The obtained black precipitate was separated from the liquid phase using a magnetic field, then magnetically washed thrice with ethanol (70% v/v) twice with phosphate-buffered saline (PBS, 100 mM sodium phosphate, 150 mM NaCl, pH 7.4). The MNPs were dried for 1 week at 37 °C in ethanol. With this protocol, the size of the nanoparticles is around 20 nm^49^ before clustering for the purpose of the current study.

### 4.2 Preparation of Electromagnetic Nanofibers Via Coaxial Electrospinning

Fibers with a magnetic core and a mechanically resistant shell were obtained using coaxial electrospinning. The setup involved a core solution with 10% (w/v) Gelatin type B (Sigma-Aldrich, Mw 360.000 g·mol^–1^) and 1% MNPs dissolved in hexafluoropropylene (HFP, MilliporeSigma) at 60 °C to avoid natural gelation of the solution during the electrospinning deposition, and a shell solution of polycaprolactone (PCL, 13% w/v, Sigma-Aldrich Mw 80,000) dissolved in HFP (MilliporeSigma) at 60 °C, the fibers were deposited in a copper collector with parallel bars to obtain aligned fibers. The optimal electrospinning conditions included a 21G needle, a distance of 10 cm between the needle and the collector, a flow rate of 1.0 mL·h^–1^, and a potential of 17.5 kV. Finally, the fibers were immersed in an aqueous solution of PEDOT:PSS (Clevios PH 1000) with 3% of Divinylsulfone (DVS V3700, Sigma-Aldrich) and incubated for 1 h at 50 °C.

### 4.3 Preparation of Electromagnetic Patterned Hydrogels

Preparation of biocompatible hydrogels mixed with conductive and magnetic compounds.

Ferrogels made of Gelatin type B (Sigma-Aldrich, 8% w/v, Mw 360.000 g·mol^–1^) containing 3% MNPs were mixed in Milli-Q water at 60 °C and later cross-linked with glutaraldehyde (Sigma-Aldrich) while exposed to a magnetic bar with an intensity of 0.5 T at a distance of 3 cm to achieve magnetic particle alignment during gelation. The cross-linked hydrogel was immersed in a solution containing PEDOT:PSS with 3% (v/v) DVS and annealed by thermal exposure for 1 h at 50 °C. A PEDOT:PSS film was also fabricated using the same procedure of the PEDOT:PSS coating for the scaffolds.

### 4.4 Scaffolds Characterization: Scanning Electron Microscopy (SEM)

The morphology and cross-section of the electrospun fibers were analyzed using a field-emission scanning electron microscope (FEG-SEM) JEOL JSM-7001F (JEOL, Akishima, Tokyo, Japan) at 5 kV, and a Hitachi S-2400 SEM (Hitachi, Chiyoda, Tokyo, Japan) at 20 kV, both following coating with a thin layer of gold/palladium. The average diameter of the electrospun fibers was measured using NIH ImageJ software (National Institutes of Health, MD). Additionally, hydrogel samples were observed using a FEI ESEM Quanta 600 (Thermo Fisher Scientific, Waltham, MA) at an acceleration voltage of 5 kV, without the need for coating.

### 4.5 Scaffolds Characterization: Transmission Electron Microscopy (TEM)

To assess variations in density within the electrospun fibers due to the magnetic core, coaxial electrospun fibers were observed using transmission electron microscopy (TEM) (HITACHI H-8100, 200 kV, LaB6 filament).

### 4.6 Scaffolds characterization: Differential scanning calorimetry (DSC)

Differential scanning calorimetry (DSC) analysis of the scaffolds was conducted using a Netzsch DSC-200-F3Maia (Netzsch Holding, Selb, Germany). The DSC measurements were performed at a heating rate of 5 °C min^–1^, starting from −30 to 100 °C for two cycles, followed by a final cycle of 420 °C.

### 4.7 Scaffolds Characterization: Attenuated Total Reflectance-Fourier Transform Infrared Spectroscopy (ATR-FTIR)

The chemical characterization of the scaffolds was performed using a Spectrum Two FTIR Spectrometer (PerkinElmer, Waltham, MA), equipped with a Pike Technologies MIRacle ATR accessory. Transmittance spectra were determined in the range of 400 to 4000 cm^–1^ with a resolution of 4 cm^–1^ and 8 scans at room temperature. Automatic baseline correction was applied using the acquisition software.

### 4.8 Scaffolds Characterization: Four-Point Probe Electroconductivity Measurement

Four 50 nm-thick gold stripes with 1 cm width were deposited onto the scaffolds using a thermal evaporation system (Edwards Coating System E 306A, Edwards, Irvine, CA) to enhance electrical contact with the measurement equipment. The electroconductivity of the different scaffolds was measured using the four-point probe method, with a Keithley DC power source (Keithley Instruments, Cleveland, OH) and an Agilent 34401A multimeter (Agilent Technologies, Santa Clara, CA). The thickness of the scaffolds was measured using a caliper (from 200 to 500 μm).

### 4.9 Scaffolds Characterization: Contact Angle

The magnetic responsiveness of the scaffolds was evaluated by measuring the surface contact angle under exposure to a magnetic field. A glycerol droplet was applied to the scaffold surface, and the contact angle was measured using the sessile drop technique, both in the presence and absence of a magnet. The droplet was recorded with a Keyence microscope (20× objective) at a 90° angle relative to the sample surface.

### 4.10 Scaffolds Characterization: Mechanical Properties

Compression mechanical testing of the hydrogels (1 × 1 cm) was conducted using an Instron Tensile Compression system at Elastomer Technology, with a 100 N load. The same equipment was modified to perform uniaxial tensile tests on the electrospun fiber scaffolds, using 50 N tensile grips. The fiber scaffolds were cut into rectangular strips (40 mm × 10 mm, *n* = 5). Young’s modulus was determined from the linear region of the stress–strain curve between 0–15% strain, while the ultimate tensile strength and maximum extension were calculated from the peak of the stress–strain curve.

### 4.11 Scaffolds Characterization: Thickness and Surface Properties

The thickness, surface profile, and roughness of the scaffolds were measured using white light interferometry (Sensofar S Neox) with 20× and 50× objectives (the latter used for the electrospun fibers).

### 4.12 Scaffolds Characterization: MTT Cytotoxicity Assay

The biocompatibility of the scaffolds was demonstrated through cytotoxicity assays.

Indirect assay was performed using latex material as the positive control and fibroblast culture media as the negative control. The lixiviates of the scaffolds incubated for 48 h were used as a replacement for the fibroblasts culture media and incubated for another 24 h. MTT solution (1 mg·mL^–1^, ThermoFisher) was prepared and replaced the lixiviates in the fibroblasts culture, in a 2 h incubation period. MTT solvent (HCl and isopropanol – 1:100, Sigma-Aldrich) was added to MTT solution in the cell culture and stirred for 5 min. Absorbance was quantified at 570 nm to determine cell metabolic activity.

Contact test: Fibroblasts (NHDF cell line) were seeded at 80,000 cells·cm^–2^ in a 24 well plate and incubated for 48 h with low-glucose Dulbecco’s Modified Eagle Medium (DMEM, Gibco, Grand Island, New York) supplemented with 10% fetal bovine serum (Gibco) and 1% antibiotic-antimycotic (Gibco) and kept at 37 °C, 5% CO_2_ and 21% O_2_ in a humidified atmosphere. Sterile scaffolds were seeded on the fibroblast monolayer for 24 h and then were observed in the optical microscope for quantification of the halo formed in the area between the scaffold and the monolayer.

### 4.13 Fibroblasts Culture on Scaffolds

The human dermal fibroblasts cell line (NHDF) were cultured using high-glucose Dulbecco’s Modified Eagle Medium (DMEM, Gibco, Grand Island, New York) supplemented with 10% fetal bovine serum (FBS, Gibco), 1% Penicilin/Streptomycin, 25 mM HEPES, 4.5 g·L^–1^ d-glucose, and l-glutamine. Cells were maintained in an incubator at 37 °C, with 5% CO_2_ and 21% O_2_ in a humidified atmosphere. Medium renewal was performed every 3–4 days. Cells were seeded on the scaffolds (PEDOT:PSS-coated coaxial fibers scaffolds, coaxial fibers, conductive hydrogel, magnetically aligned hydrogel and PEDOT:PSS film) at a density of 150,000 cells on cell culture plates with a covalently bound hydrogel layer to inhibit cellular attachment (Costar 24 ultralow attachment well plates). On day 6 after seeding the cells on the hydrogels, the fibroblasts were stained using Calcein AM to observe cell viability on the scaffolds.

### 4.14 iPSC-CM Differentiation and Seeding on the Scaffolds

A human induced pluripotent stem cell (hiPSC) line – the Gibco episomal hiPSC line (Thermo Fisher Scientific) was derived from CD34+ cord blood through EBNA-based episomal transfection of factors SOX2, OCT4, KLF4, C-MYC, NANOG, LIN28, and SV40 T antigen.^61^ The procedures for isolation, culture, differentiation, and dissociation are described elsewhere.^46,52^ Briefly, early iPSC-CMs were seeded onto 6-well plates. Dissociation of the cells occurred 6–8 weeks after differentiation, and they were seeded onto the plates at an approximate concentration of 2 million cells·mL^–1^ in basal RPMI medium (Life Technologies, 11875093), supplemented with 1× B27 (Life Technologies, 17504044), the Y-27632 ROCK inhibitor (1:1000; Tocris, 1254), and 10% fetal bovine serum. The medium was replaced after 24 h with RPMI/B27, and cells were incubated for an additional 2 weeks before further experiments were conducted on the electroconductive scaffolds. iPSC-CMs were seeded on the scaffolds at a density of 250.000 per cm^2^. During this period, the cells exhibited a consistent beating frequency of 22.5 beats per minute (cardiac frequency 0.35 Hz; Fc = HR × 0.016667), which was consistent across all wells. The purity of iPSC-derived cardiomyocytes (iPSC-CMs) after differentiation was assessed by immunostaining with a monoclonal primary antibody against cardiac Troponin I (Sigma-Aldrich, WH0007137M4), diluted 1:1000 in PBS (Figure S5A). iPSC-CMs were fixed in 4% formaldehyde (VWR) and permeabilized with 0.1% Triton X-100 (Sigma-Aldrich). Nonspecific binding was blocked by incubating the cells with 4% fetal bovine serum (FBS) (Sigma-Aldrich) in PBS for 1 h at room temperature, followed by overnight incubation at 4 °C. Samples were washed with 0.05% Tween-20 and then incubated with a rabbit antimouse Alexa Fluor 488 secondary antibody (Invitrogen, 1:500) for 2 h. Additionally, the tools MuscleMotion and Myocyter used to assess CMs beating detected 80% movement within the area of all videos assessed, further contributing to the determination of the purity and functionality of the differentiated CMs (Supporting Information).

### 4.15 Cell Viability of iPSC-CMs On-Chip

Viability studies were performed after seeding the CMs within the organ-on-chip platform. A control condition, consisting of iPSC-CMs seeded on PDMS (400.000 cells·mL^–1^), was used for comparison. iPSC-CMs were washed with complete PBS (Sigma-Aldrich) and incubated for 30 min in 1 μM acetoxymethyl (AM) Calcein solution (Sigma-Aldrich, C1359) in PBS to stain viable cells. Dead cells were stained with 5 μM ethidium homodimer I (Sigma-Aldrich, E1903) in PBS. Fluorescence images were captured using an inverted EVOS FL fluorescence microscope.

### 4.16 Metabolic Activity of iPSC-CMs on Scaffolds

The metabolic activity of iPSC-CMs was assessed using AlamarBlue cell viability reagent (Molecular Probes, Eugene, OR) on days 3 and 8 of the culture, seeding 20.000 iPSC-CMs per cm^2^ on each scaffold. AlamarBlue reagent was added to the cells and incubated for 2 h at 37 °C in a 5% CO_2_ incubator. Fluorescence was measured within the wavelength range of 560–590 nm. Prior to analysis, a calibration curve was generated for varying iPSC-CMs density (10,000, 50,000, 100,000, 150,000 cells·mL^–1^) to correlate cell count with metabolic activity, following manufacturer’s instructions. This calibration curve was used to convert the measured metabolic values and obtain proliferation curves for each condition.

### 4.17 Microchip Setup & Electrical and Magnetic Stimulation

A PDMS (Dow Corning) construct with a geometry consisting of two cell compartments and a central region for coculturing was fabricated using soft lithography procedures, as previously described.^13^ The construct was then bonded to a 24-well plate with the geometry facing upward. Electromagnetic scaffolds were cut into pieces matching the size of the central coculture region and securely attached to the PDMS using Medical Adhesive Type A (Biesterfeld). The images of the PDMS construct with the electrical setup can be found in Figure S7. The constructs were sterilized using a combination of 30 min of UV exposure followed by 24 h incubation in PBS containing 1% antibiotic-antimycotic. Prior to cell seeding, the samples were incubated in cell culture medium for 4 h. iPSC-derived cardiomyocytes (iPSC-CMs) and human umbilical vein endothelial cells (HUVECs) were cocultured on top of the microchips containing electromagnetic scaffolds (comprising coaxial PCL/Gelatin/PEDOT:PSS fibers and Gelatin/PEDOT:PSS hydrogel) and electrically stimulated using a custom-built electrode setup, as described previously.^52^ Briefly, platinum electrodes were inserted into a 24-well plate and secured with silicone adhesive. Electrical connections were made to the electrodes, which were connected to a programmable AC/DC power source (EC1000SA, NF Corporation), and the electrical joints were isolated to prevent environmental interference. A pulsed electric field with a monophasic sine wave was applied using an external power supply, with an AC potential ranging from 0 to 10 V at 3 Hz for 24 h.

### 4.18 Coculture of iPSC-CMs and HUVECs

For this experiment, the organ-on-chip platform was preincubated with iPSC-CM culture medium for 24 h prior to cell seeding. On day 1, iPSC-CMs were introduced into the chip at a high density of 1,000,000 cells and incubated for 48 h. On day 3, HUVECs were added into the chip at a density of 2,500,000 cells. At this stage, the culture medium was replaced with a 1:1 mixture of HUVEC and iPSC-CM media. On day 6, combined electrical and magnetic stimulation was applied to the chip for 24 h using the parameters detailed in Section 4.17. The experiment was terminated on day 7, at which point the cocultures were subjected to analysis.

### 4.19 Immunostaining of iPSC-CMs and HUVECs

iPSC-CMs were initially immunostained by following the protocol described in Section 4.14 in an independent step to minimize cross-reactivity. Immuno-characterization of HUVECs was performed after immunostaining of iPSC-CMs using a CD31 antibody after coculture (iPSC-CMs and HUVECs) fixation with 4% paraformaldehyde and blocking with 10% fetal bovine serum (FBS, Gibco) in PBS. The primary antibody against CD31 (1:50 dilution, mouse monoclonal, Dako) was applied in the blocking solution and incubated overnight. The secondary antibody, Alexa Fluor 647 (1:500 dilution, goat antimouse, Abcam), was then added and incubated for 30 min. Fluorescence images were captured using a Leica DM IL LED fluorescence microscope with an EC3 camera system.

### 4.20 iPSC-CMs Beating Assessment

The single-cell beating behavior of iPSC-CMs was analyzed using the MuscleMotion and Myocyter Fiji plugins, based on video recordings of the iPSC-CMs and iPSC-CMs-HUVECs cultures.

### 4.21 Statistical Analysis

All measurements were performed in triplicate under independent conditions. Results are presented as the mean ± standard deviation (SD). A two-way ANOVA with Sidak’s multiple comparisons test was used to compare the means of three independent measurements (*n* = 3). Statistical analysis was conducted using GraphPad Prism version 7 (GraphPad Software, La Jolla, CA). *p < 0.05 was considered statistically significant; **p < 0.01, very significant; ***p < 0.001, highly significant; and ****p < 0.0001, extremely significant.

## Supplementary Material









## Notes

Beating profile of differentiated cardiomyocytes cultures on day 11 (MP4)
Beating profile of differentiated cardiomyocytes cultures on day 21 (MP4)
Differential scanning calorimetry (DSCs) results for all tested scaffolds; additional SEM images for the fibers and magnetic nanoparticles (MNPs); additional TEM images for the coaxial fibers; maximum fiber elongation during tensile mechanical testing; purity and beating parameters of iPSC-CMs using Myocyter analysis; biocompatibility assay for fibers and hydrogels; images of electrical stimulation setup (PDF)
Beating profile of a co-culture of HUVECs and cardiomyocytes-on-chip (MP4)
