# Supplementary material for: Biomimetic Model for Electromagnetic Modulation of Cardiovascular Cellular Interactions On-Chip
Source: ACS Appl Bio Mater. 2025 Jul 16;8(8):7080–94. doi: 10.1021/acsabm.5c00798 (PMC12365882; doi:10.1021/acsabm.5c00798)
Supplement: Supplementary file 3 [file mt5c00798_si_003.pdf]

Supporting Information

Biomimetic model for electromagnetic modulation of cardiovascular cellular interactions on-chip

Ana C. Manjua[1,2*], Fábio F. F. Garrudo[3,4], Ana Agostinho[4,5,6], Afonso Gusmão[5,6,7], Paola Sanjuan-Alberte[5,6], Frederico Castelo Ferreira[5,6], Burcu Gumuscu[1,2,8*]

[1]Biosensors and Devices Lab, Department of Biomedical Engineering, Eindhoven University of Technology, Eindhoven, 5600 MB, Netherlands

[2]Institute of Complex Molecular Systems, Eindhoven University of Technology, Eindhoven, 5600 MB, Netherlands
[3]Department of Brain and Cognitive Sciences, Picower Institute for Learning and Memory, Massachusetts Institute of Technology, Cambridge, MA, USA.
[4]Instituto de Telecomunicações, Instituto Superior Técnico, Avenida Rovisco Pais, 1049-001 Lisboa, Portugal
[5]Department of Bioengineering and iBB-Institute for Bioengineering and Biosciences, Instituto Superior Técnico, Universidade de Lisboa, Av. Rovisco Pais, 1049-001, Lisbon, Portugal
[6]Associate Laboratory i4HB—Institute for Health and Bioeconomy, Instituto Superior Técnico, Universidade de Lisboa, Av. Rovisco Pais, 1049-001, Lisbon, Portugal
[7]IDMEC, Instituto Superior Técnico, Universidade de Lisboa, Av. Rovisco Pais, 1049-001, Lisbon, Portugal
[8]Eindhoven Artificial Intelligence Systems Institute, Eindhoven University of Technology, Eindhoven, 5600 MB, Netherlands

***Corresponding authors:**
Ana C. Manjua: a.c.baeta.manjua@tue.nl
Burcu Gumuscu: b.gumuscu@tue.nl

Table S1. Differential scanning calorimetry (DSCs) results for all tested scaffolds.

Samples	Aligned Hydrogel	Conductive Hydrogel	Coaxial Fibers	Conductive Fibers	PEDOT:PSS film	PCL Fibers	Gelatin
Cycle 1	Peak: 55.2°C *1	Peak: 76.8°C *2	Peak 1: 69.5°C *3<br><br>Peak 2: 69.4°C	Peak: 57.2°C *4	Peak 1: 61.5°C	Peak 1: 62.4°C	Peak 1: 58.3°C
Cycle 2	No Peak	No Peak	Peak: 32.8°C	Peak: 33°C	No Peak	Peak: 26.3°C	No Peak
Cycle 3	No Peak	No Peak	Peak: 56.3°C	Peak: 55.7°C	No Peak	Peak: 57.1°C	No Peak
Cycle 4	No Peak	No Peak	Peak: 32.7°C	Peak: 32.7°C	No Peak	Peak: 26.6°C	No Peak
Cycle 5	No Peak	No Peak	Peak: 56.4°C	Peak: 55.9°C	No Peak	Peak: 57.1°C	No Peak
Cycle 6	No Peak	No Peak	Peak: 32.8°C	Peak: 32.6°C	No Peak	Peak: 26.8°C	No Peak

*1

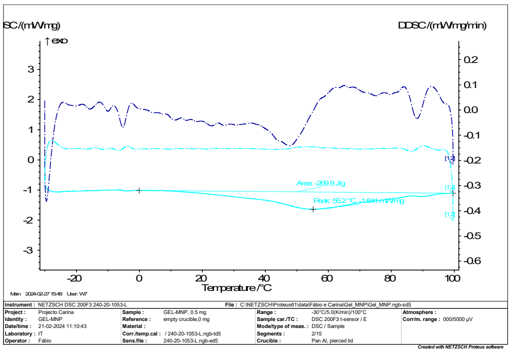

*2

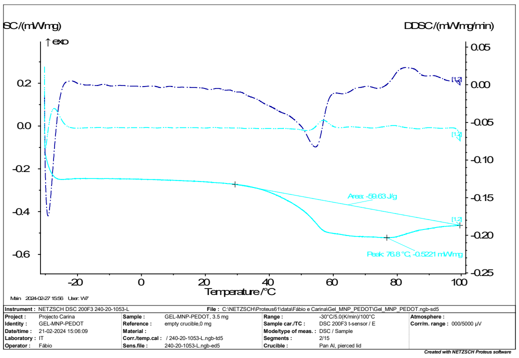

*3

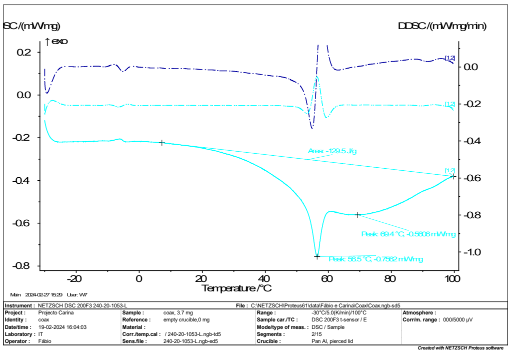

*4

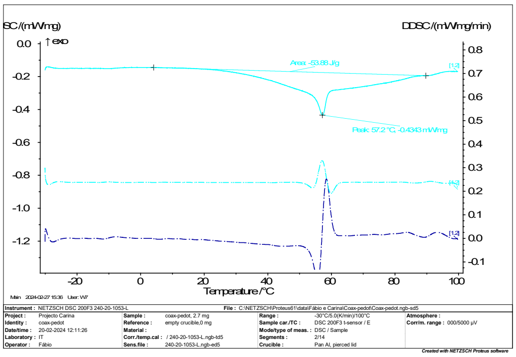

Figure S1. Representative DSC thermograms for the cycle 1 of Aligned hydrogel (*1), Conductive Hydrogel (*2), Coaxial fibers (*3) and Conductive fibers (*4).

A

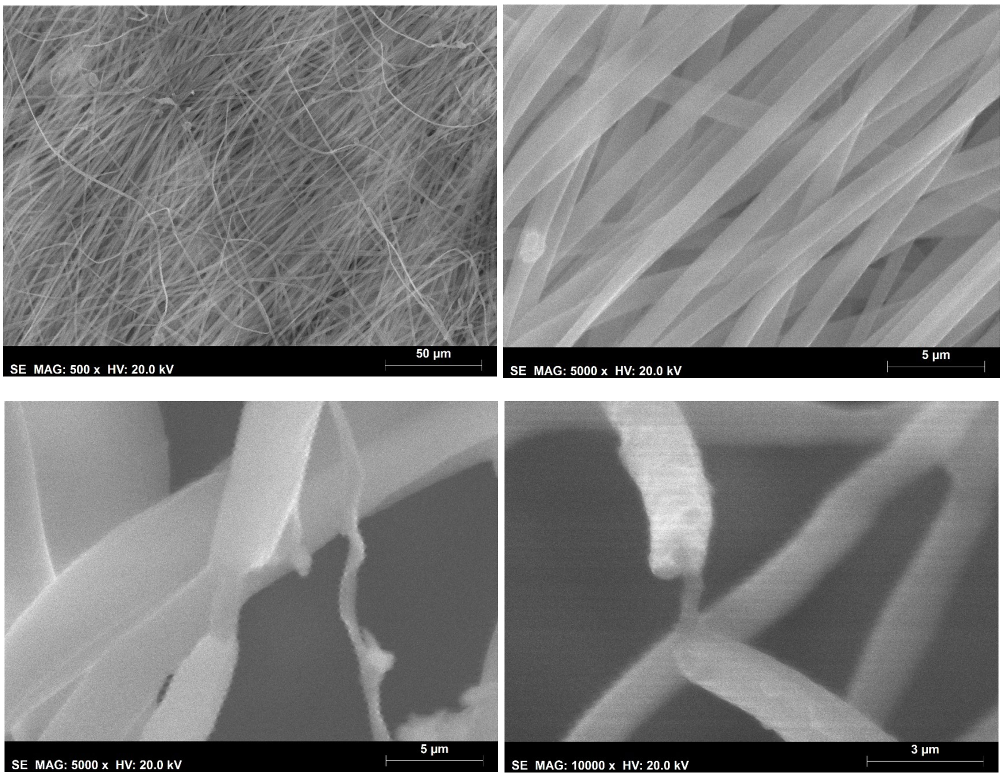
50 µm
SE MAG: 500 x HV: 20.0 kV
5 µm
SE MAG: 5000 x HV: 20.0 kV
5 µm
SE MAG: 5000 x HV: 20.0 kV
3 µm
SE MAG: 10000 x HV: 20.0 kV

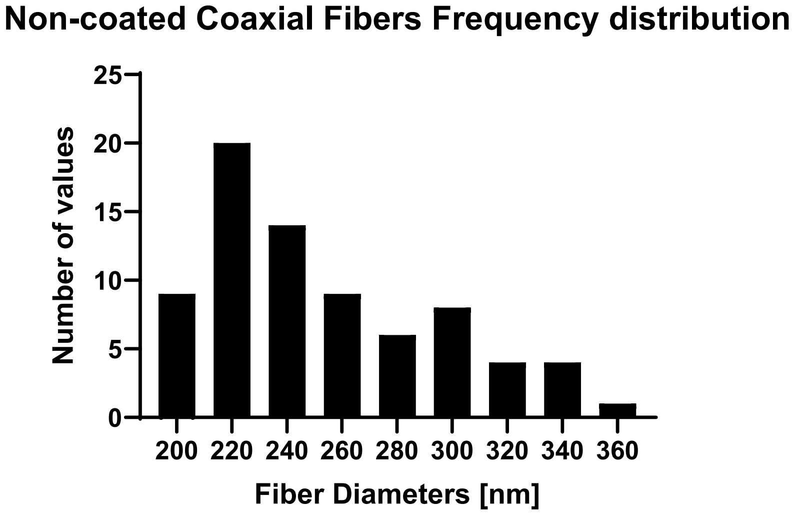
Non-coated Coaxial Fibers Frequency distribution
Number of values
0
5
10
15
20
25
200 220 240 260 280 300 320 340 360
Fiber Diameters [nm]

B

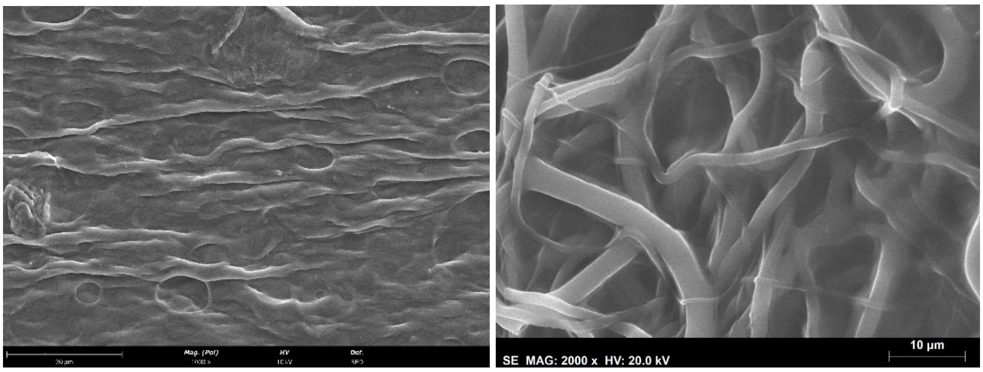
SE MAG: 2000 x HV: 20.0 kV
10 µm

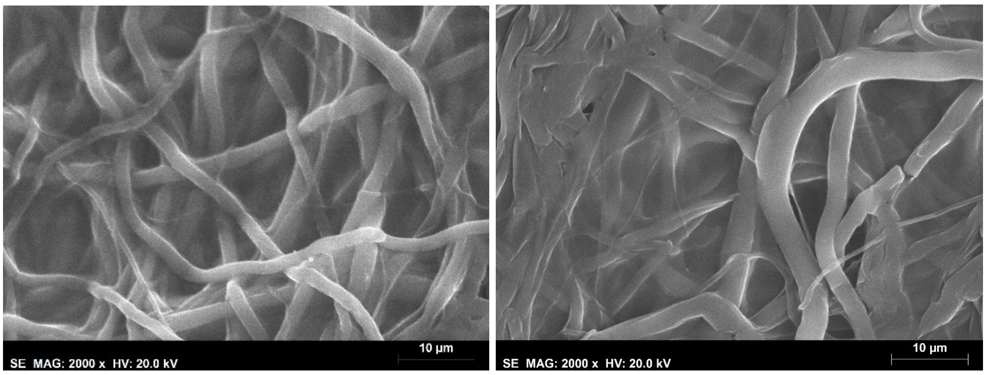

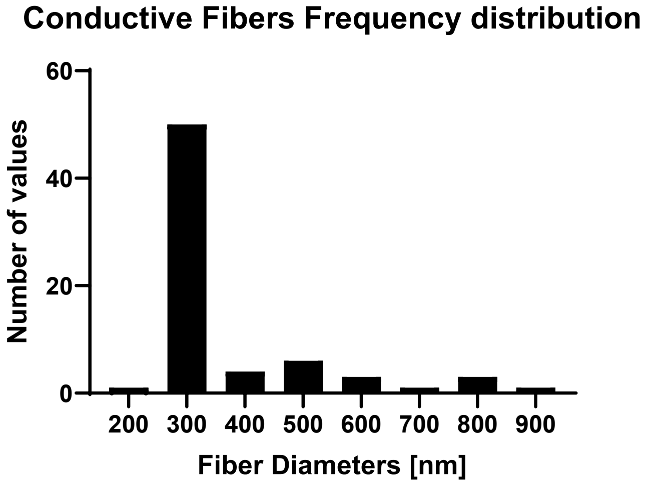

C

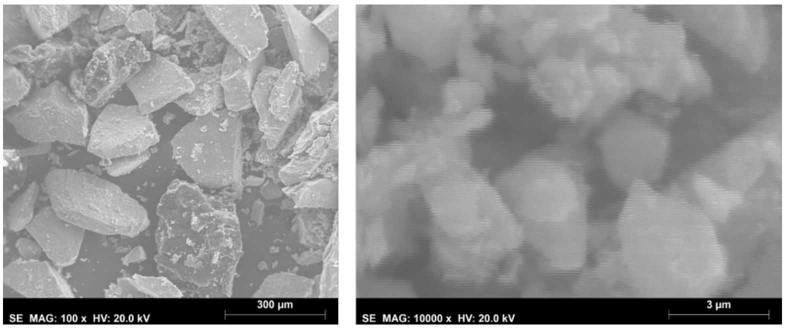

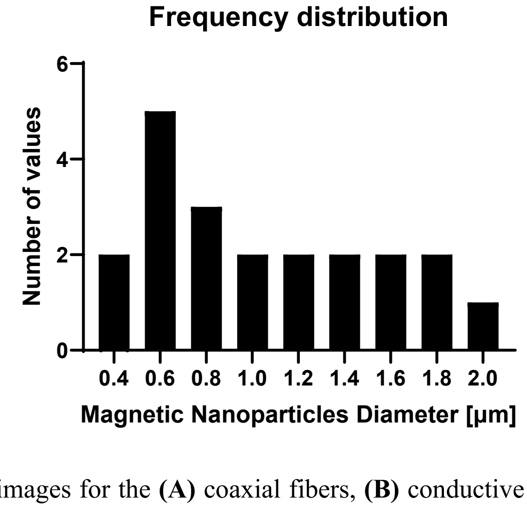

Figure S2. Additional SEM images for the **(A)** coaxial fibers, **(B)** conductive (PEDOT: PSS-coaxial) fibers and (**C**) magnetic nanoparticles (MNPs).

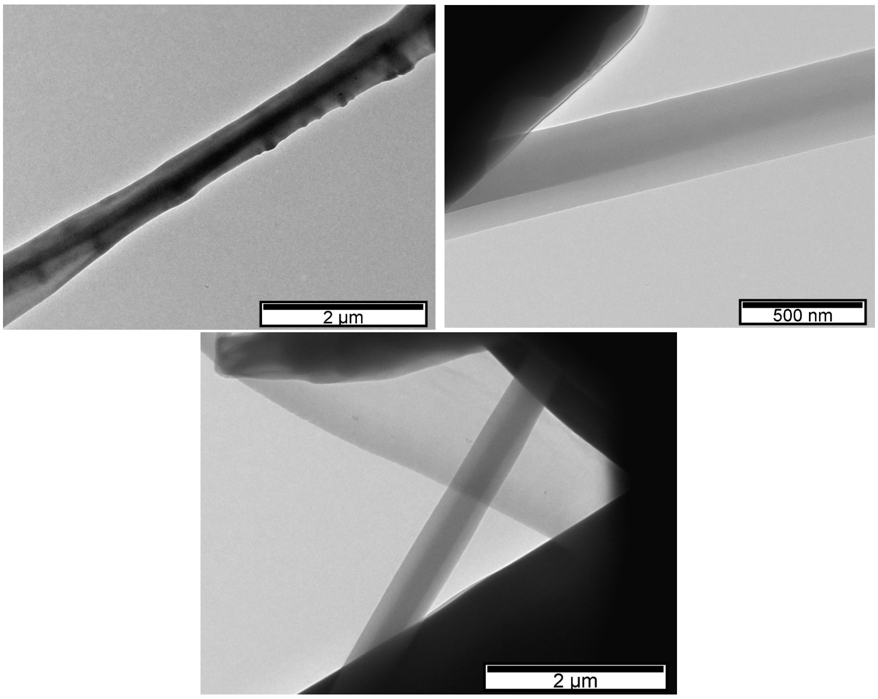

Figure S3. Close-up TEM images for coaxial fiber characterization.

A **B**

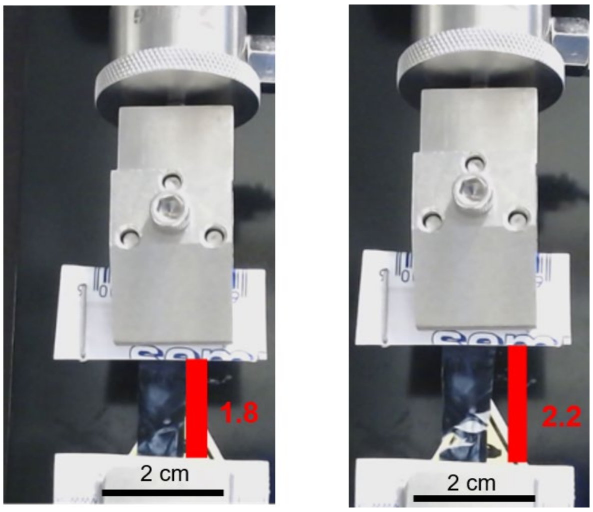

Figure S4. PEDOT: PSS-coaxial fibers before **(A)** and at maximum elongation **(B)** during tensile mechanical testing.

Table S2. Assessment of beating parameters of iPSC-CMs two weeks after differentiation using Myocyter analysis.

Cardiomyocytes Analysis	Min	Max	Mean	StdDev
Beat times [sec]	0,45	1,55	0,9944	0,382
Frequency [1/sec]	0,6452	22.222	11.648	0,5021
Amplitudes [a,u,]	0,8839	33.917	23.446	11.918
Peaktimes user [sec]	0,3	0,85	0,5429	0,1644
Systoles user [sec]	0,1	0,65	0,3	0,1732
Diastoles user [sec]	0,1	0,35	0,2214	0,07559

A.1. iPSC-CMs 10 days after differentiation

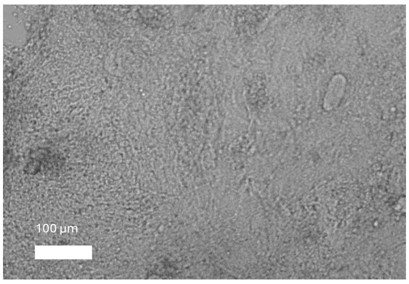

A.2. iPSC-CMs Purity assessment: 73.6%

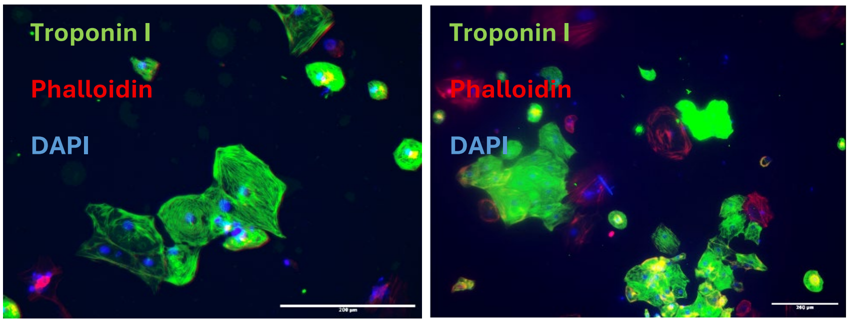

A. Amplitudes of the contractility of iPSC-CMs using Myocyter

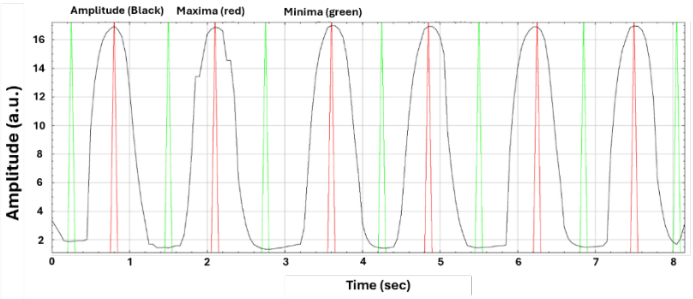

B. Amplitudes of the contractility of iPSC-CMs using MuscleMotion

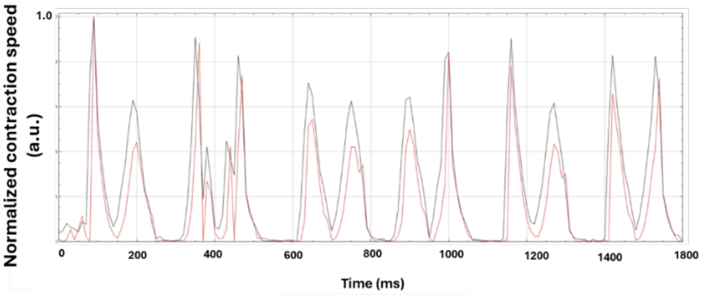

Figure S5. **(A.1)** Additional iPSC-CMs brightfield imaging 10 days after differentiation (day 11), **(A.2)** Purity assessment of the iPSC-CMs differentiation. **(B)** contractility (amplitudes) profile using Myocyter and **(C)** normalized contractility speed results using Musclemotion ImageJ plugins.

A1. MTT Assay Results.

Samples (MTT assay)	Aligned Hydrogel	Conductive Hydrogel	Coaxial Fibers	Conductive Fibers	PEDOT: PSS film	Positive Control (Media)	Negative Control (Latex)
Viability (%)	85	73	158	99	62	100	54

A2. Direct contact assay

Positive Control

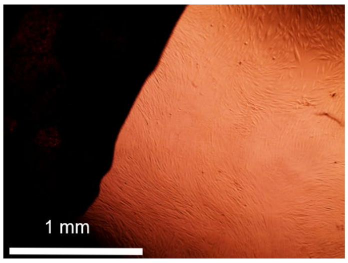

Negative Control

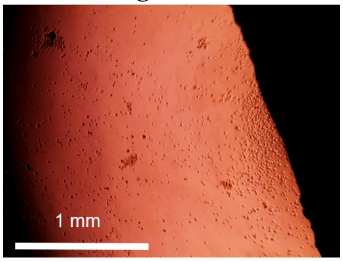

Coaxial Fibers

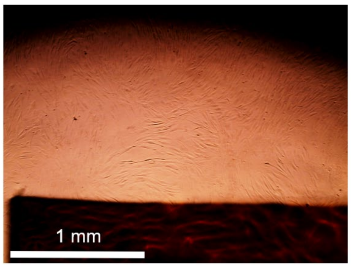

Conductive Fibers

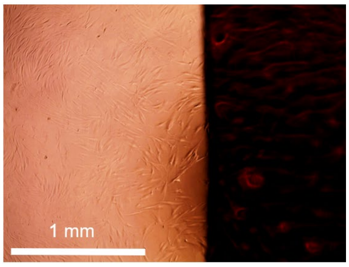

Aligned Hydrogel

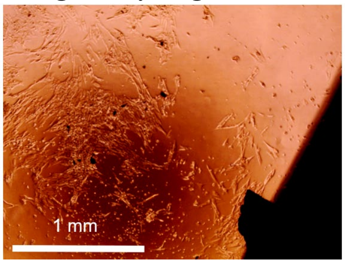

Conductive Hydrogel

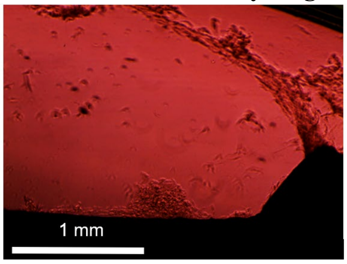

PEDOT: PSS film

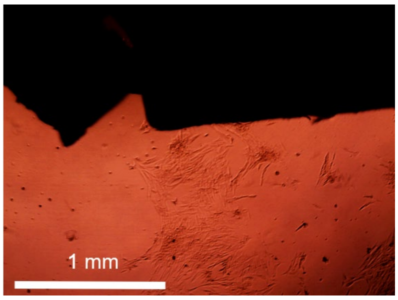

B. Fibroblasts viability in Hydrogel (aligned hydrogel and conductive hydrogel)

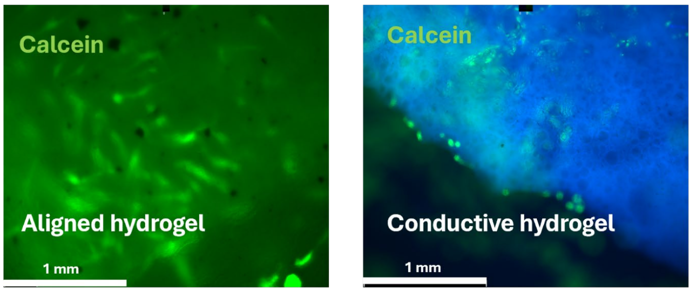

Figure S6. A. Cytotoxicity results for aligned hydrogel and conductive hydrogel (**A1.** MTT assay, normalized for the positive control - cell culture media and **A2.** direct contact) using NHDF. **B.** Biocompatibility of the aligned hydrogel and conductive hydrogel using NHDFibroblasts.

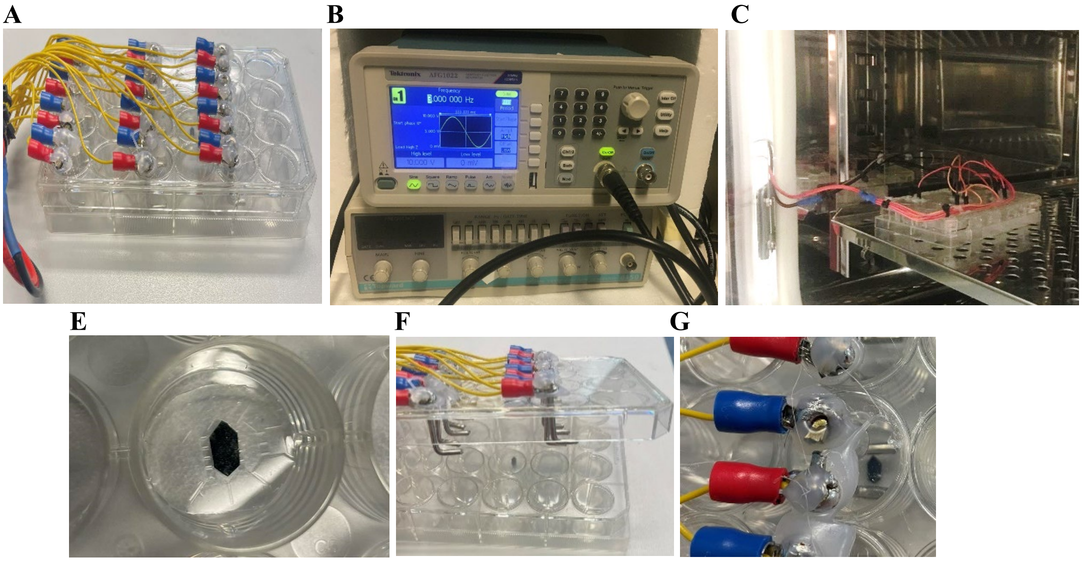

Figure S7. Electrical stimulation setup. **(A)** Plate lid with electrodes for electrical stimulation in a 24-well plate. **(B)** Power source configuration. **(C)** Electrical stimulation of cardiomyocytes co-culture for 24h. **(D)** PDMS bonded to the bottom of a 24-well plate prior to electrical stimulation. **(E)** Plate lid with electrodes connecting to the PDMS device. **(F)** The positioning of the electrodes aligns with the conductive scaffold.

A. Single Magnetic Stimulation

Region 1 **Region 2** **Region 3 (Middle area)**

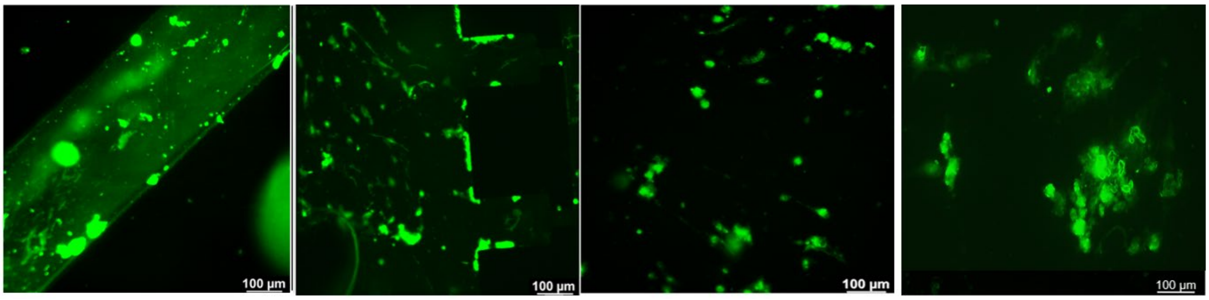

B. Single Electrical Stimulation

Region 1 **Region 2** **Region 3 (Middle area)**

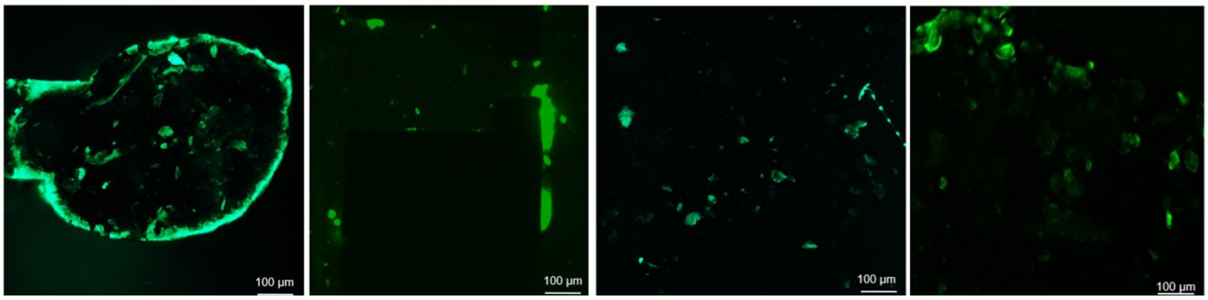

Figure S8. Controls for electromagnetic stimulation for the three different regions analyzed. Single Magnetic stimulation in **(A)** and single Electrical stimulation in **(B)**.

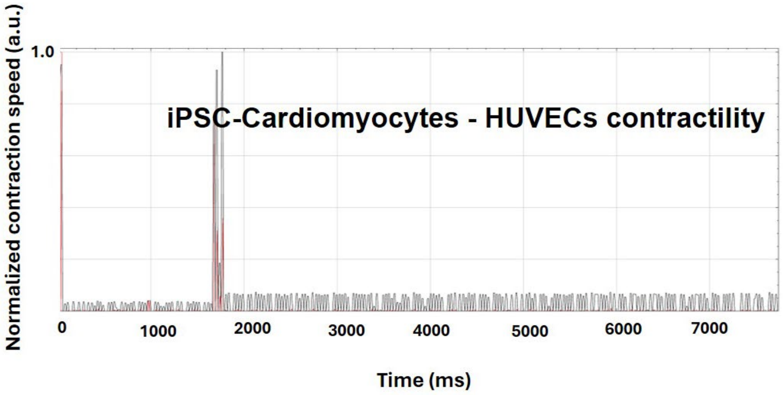

Figure S9. Contractility profile of the co-culture of iPSC-CMs and HUVECs in the middle region of the chip using the MuscleMotion tool.

Electronic Supporting data.

Movie1. Available video of the beating profile of differentiated cardiomyocytes cultures at day 11.
Movie2. Available video of the beating profile of differentiated cardiomyocytes cultures at day 21.
Movie3. Available video of the beating profile of a co-culture of HUVECs and cardiomyocytes on chip.
